# Tumor-colonizing *Pseudoalteromonas elyakovii* metabolically reprograms the tumor microenvironment and promotes breast ductal carcinoma

**DOI:** 10.1128/mbio.03873-24

**Published:** 2025-04-07

**Authors:** Shuyan Liu, Youpeng Pan, Chaopeng Zheng, Qinghui Zheng, Yaoqiang Du, Yajuan Zheng, Hongchao Tang, Xiaozhen Liu, Jiancheng Mou, Xin Zeng, Zhuotao Yang, Wenjuan Gui, Yuning Tang, Mingxing Xu, Zhihao Ye, Haotian Su, Qiuran Xu, Xuli Meng

**Affiliations:** 1 Department of Breast Surgery, General Surgery, Cancer Center, Zhejiang Provincial People's Hospital (Affiliated People's Hospital), Hangzhou Medical College 723615, Hangzhou, Zhejiang, China; 2 Key Laboratory for Diagnosis and Treatment of Upper Limb Edema and Stasis of Breast Cancer, Hangzhou, Zhejiang, China; 3 The Second Clinical Medical College, Zhejiang Chinese Medical University 587400, Hangzhou, Zhejiang, China; 4 Laboratory Medicine Center, Department of Transfusion Medicine, Zhejiang Provincial People’s Hospital (Affiliated People’s Hospital), Hangzhou Medical College 74678, Hangzhou, Zhejiang, China; 5 The Second School of Clinical Medicine, Hangzhou Normal University, Hangzhou, Zhejiang, China; 6 Wenzhou Medical University, Hangzhou, Zhejiang, China; 7 Zhejiang Key Laboratory of Tumor Molecular Diagnosis and Individualized Medicine, Zhejiang Provincial People's Hospital, Affiliated People's Hospital, Hangzhou Medical College 578309 , Hangzhou, Zhejiang, China; Louis Stokes Veterans Affairs Medical Center, Cleveland, Ohio, USA

**Keywords:** breast cancer, intratumoral microbiota, *Pseudoalteromonas elyakovii*, tumor microenvironment

## Abstract

The correlation between the microbiota found in tumors and tumor development is being progressively understood, specifically regarding its involvement in the initiation and advancement of tumors. We examined a total of 102 samples, examining the microbial composition at the species level in each person unveiled significant variations in both the microbial makeup and tumor proportions among individuals, examining the fluctuating alterations in the microbial profile during breast cancer advancement and progression. The levels of expression for *Pseudoalteromonas elyakovii* were notably elevated in the tumor groups when compared to the para-cancer normal group, aligning with the results obtained from qRT-PCR analysis. The relationship between tumor immunity and microorganisms within the tumor was investigated using double immunofluorescence staining combined with SweAMI probe *in situ* hybridization and scRNA-seq, allowing for an in-depth analysis of intratumoral microorganisms. Experiments have demonstrated that the supernatant derived from *P. elyakovii* displayed a significant ability to promote tumor growth and stimulation. In summary, we describe the characteristics of the intratumoral microbiota and the tumor-promoting effects of *P. elyakovii* supernatant within a small dose range in ductal carcinoma of the breast and characterize the potential clinical application value of intratumoural microorganisms in the progression of cancer and immunotherapy.

**IMPORTANCE:**

Despite the existing studies, the specific microbial factors that influence the occurrence and progression of breast cancer still remain unclear. Researchers have clarified the distinctive microbial profile related to ductal carcinoma, a common histological type of breast cancer, in order to identify tumor-specific microbes and their roles in tumorigenesis. With the tumor microbiome as the focus, the enrichment of *Pseudoalteromonas elyakovii* features accelerates the disease progression in patients with ductal carcinoma of the breast. This study reveals the initial role relationship and innovative findings between *Pseudoalteromonas elyakovii* and ductal carcinoma in the breast.

## INTRODUCTION

Breast cancer (BC), a malignant neoplasm arising from the breast’s epithelial cells, has been identified as the globe’s second most prevalent cancer form. On 1 February 2024, the IARC, which is affiliated with the WHO, announced that the global number of new breast cancer cases in 2022 was projected to be around 2.3 million (1). Traditionally, tumors and microbial infections were viewed as distinct conditions; nevertheless, the emergence of advanced sequencing technology has transformed our comprehension of the interaction between microorganisms and tumorigenesis (2). The gut microbiota holds great potential for revolutionizing cancer treatment, as scientists are devoted to investigating a comprehensive strategy that integrates microbiome regulation therapy with biological, immune, cellular, and surgical approaches in the battle against cancer (3).

With the advancement of *in vivo* microbial investigation, previously presumed sterile tissues and organs such as the lung, mammary gland, kidney, prostate, pancreas, and liver have been revealed to harbor minimal-biomass microbial communities (4, 5). Concurrently, these microorganisms exert a substantial influence on the presence and progression of tumors (6–10). Furthermore, intratumoral microflora exhibit notable variations and heterogeneity even within tumors of the same type (11, 12). Researchers hypothesize that microbiota may exert an influence on tumor tissues or other areas through diverse pathways, including metabolites and the immune system (13). A comprehensive analysis of microbiota composition can enhance our comprehension of their association with cancer biology (14). Breast cancer exhibits the highest bacterial diversity and abundance compared to other types of cancer, potentially exerting an influence on the biological characteristics of breast tumors (12, 15, 16). A significant discrepancy in intratumoral microbial load levels was observed between tumor and adjacent tissues. *Methylobacterium radiotolerans* and *Sphingomonas yanoikuyae* exhibited higher prevalence within neoplastic breast tissue relative to healthy tissue (17). Second, the presence of intratumor microbiota may play a significant role in breast cancer development. A separate study investigating the DNA of breast microbiota revealed that tumor tissues exhibited relatively higher levels of *Lactobacillus*, *Streptococcus*, and *Staphylococcus*. Conversely, paired normal tissues demonstrated a relatively higher abundance of *Sphingomonas yanoikuyae* bacteria. These findings strongly indicate a correlation between dysbiosis of microbiota and the occurrence of BC (18, 19). Third, there exists a distinct microbial interaction within the tumor microenvironment that exerts an influence on the immune system. Tzeng et al. reported a reduced abundance of certain bacteria such as *Propionibacterium* and *Staphylococcus* in tumor tissues, which exhibited a negative correlation with immune signatures associated with cancer development. Conversely, *Streptococcus* and *Propionibacterium* demonstrated a positive correlation with genes involved in T cell activation (12). Additionally, there exists a variation in the tumor microbiota across distinct subtypes of BC. The prevalence of *Streptococcaceae* family is notably higher in triple-negative BC subtypes, while the increasing abundance of *Bosea* genus as the tumor progresses provides novel biological evidence for the classification and diagnosis of breast cancer (20–22). The exact microorganisms responsible for influencing the onset and progression of breast cancer are yet to be fully elucidated based on current research findings. Despite existing studies, the specific microbial factors influencing the occurrence and progression of BC remain elusive. Researchers have elucidated the distinctive microbial profile associated with ductal carcinoma, a common histological type of breast cancer, in order to identify tumor-specific microbes and their roles in tumorigenesis. To investigate the function of microbiota within the tumor in the heterogeneity of breast ductal carcinoma. With the tumor microbiome as a focal point, researchers are dedicated to investigating complementary approaches for the identification and treatment of breast cancer across different stages. Revealing the intricate association between intratumor microorganisms and breast cancer is pivotal for the advancement of innovative therapeutic tactics.

## RESULTS

### 5R 16S rRNA sequencing revealed unique microbial presence and their specific functions in BC patients

**Fig 1:**
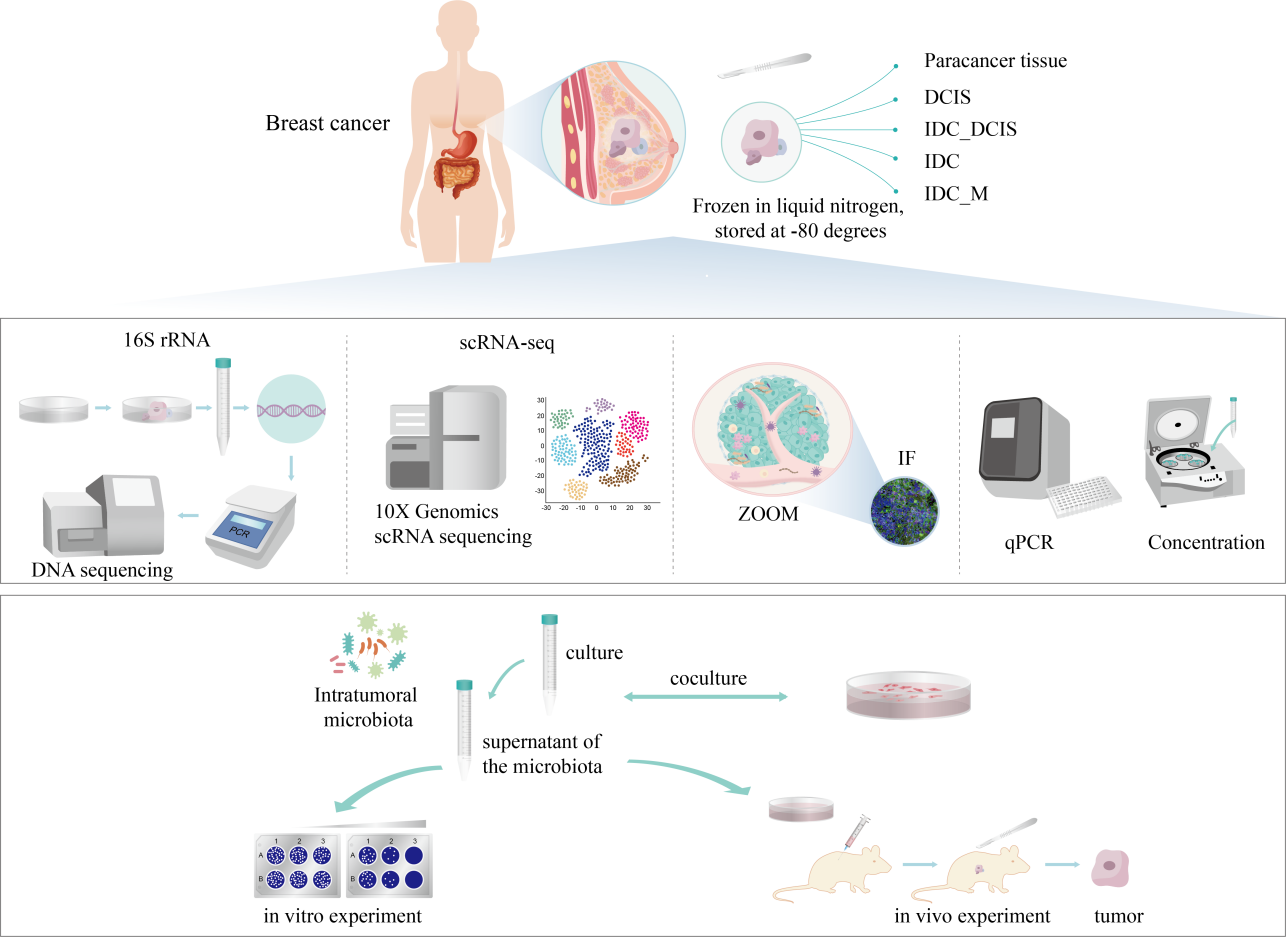
Diagrammatic depiction of the research’s overall design and the sequence of analytical procedures.

To investigate the impact of intratumor microbiota composition on the initiation and progression of ductal breast cancer in female patients, we present a comprehensive analysis of the clinical and pathological characteristics of a cohort of women diagnosed with breast malignancy who underwent mastectomy. The detailed information is provided in Table 1. This study included tumor samples and adjacent normal tissues obtained from 11 patients with breast cancer identified as having ductal carcinoma *in situ* through either biopsy or rapid intraoperative frozen pathology. Additionally, samples were collected from 80 individuals identified with infiltrating ductal carcinoma using the same diagnostic methods (Fig. 1).

**TABLE 1:** Patient demographics and clinical characteristics of BC study volunteers in the 16S sequencing study

Variable	Overall, *N* = 92^*a*^	Type				*P*-value^*b*^
		DCIS *N* = 16 (17%)^*a*^	IDC *N* = 25 (27%)^*a*^	IDC_DCIS *N* = 25 (27%)^*a*^	IDC_M *N* = 26 (28%)^*a*^	
Age, years						0.024
41–49	22 (23.91%)	9 (56.25%)	2 (8.00%)	6 (24.00%)	5 (19.23%)	
50–74	53 (57.61%)	5 (31.25%)	17 (68.00%)	17 (68.00%)	14 (53.85%)	
≤40	8 (8.70%)	2 (12.50%)	3 (12.00%)	1 (4.00%)	2 (7.69%)	
≥75	9 (9.78%)	0 (0.00%)	3 (12.00%)	1 (4.00%)	5 (19.23%)	
pT stage						<0.001
T1	43 (46.74%)	4 (25.00%)	17 (68.00%)	14 (56.00%)	8 (30.77%)	
T2	33 (35.87%)	0 (0.00%)	8 (32.00%)	10 (40.00%)	15 (57.69%)	
T3	2 (2.17%)	0 (0.00%)	0 (0.00%)	0 (0.00%)	2 (7.69%)	
T4	2 (2.17%)	0 (0.00%)	0 (0.00%)	1 (4.00%)	1 (3.85%)	
Tis	12 (13.04%)	12 (75.00%)	0 (0.00%)	0 (0.00%)	0 (0.00%)	
pN stage						<0.001
N0	53 (57.61%)	16 (100.00%)	23 (92.00%)	13 (52.00%)	1 (3.85%)	
N1	24 (26.09%)	0 (0.00%)	1 (4.00%)	5 (20.00%)	18 (69.23%)	
N2	8 (8.70%)	0 (0.00%)	1 (4.00%)	5 (20.00%)	2 (7.69%)	
N3	7 (7.61%)	0 (0.00%)	0 (0.00%)	2 (8.00%)	5 (19.23%)	
ER status						0.16
Negative	20 (21.74%)	7 (43.75%)	4 (16.00%)	4 (16.00%)	5 (19.23%)	
Positive	72 (78.26%)	9 (56.25%)	21 (84.00%)	21 (84.00%)	21 (80.77%)	
PR status						0.20
Negative	21 (22.83%)	7 (43.75%)	5 (20.00%)	4 (16.00%)	5 (19.23%)	
Positive	71 (77.17%)	9 (56.25%)	20 (80.00%)	21 (84.00%)	21 (80.77%)	
HER2 status						0.40
0+	14 (15.22%)	2 (12.50%)	3 (12.00%)	5 (20.00%)	4 (15.38%)	
1+	39 (42.39%)	5 (31.25%)	11 (44.00%)	14 (56.00%)	9 (34.62%)	
2+	22 (23.91%)	3 (18.75%)	7 (28.00%)	5 (20.00%)	7 (26.92%)	
3+	17 (18.48%)	6 (37.50%)	4 (16.00%)	1 (4.00%)	6 (23.08%)	
Ki-67 expression						0.023
15.1%–35.0%	26 (28.26%)	5 (31.25%)	4 (16.00%)	10 (40.00%)	7 (26.92%)	
≤15%	35 (38.04%)	9 (56.25%)	14 (56.00%)	7 (28.00%)	5 (19.23%)	
>35.0%	31 (33.70%)	2 (12.50%)	7 (28.00%)	8 (32.00%)	14 (53.85%)	
PAM50						<0.001
Basal-like	12 (13.04%)	3 (18.75%)	1 (4.00%)	4 (16.00%)	4 (15.38%)	
HER2	19 (20.65%)	6 (37.50%)	5 (20.00%)	1 (4.00%)	7 (26.92%)	
LumA	28 (30.43%)	5 (31.25%)	14 (56.00%)	6 (24.00%)	3 (11.54%)	
LumB	33 (35.87%)	2 (12.50%)	5 (20.00%)	14 (56.00%)	12 (46.15%)	
Histologic grade						<0.001
I	31 (33.70%)	4 (25.00%)	16 (64.00%)	10 (40.00%)	1 (3.85%)	
II	33 (35.87%)	0 (0.00%)	8 (32.00%)	8 (32.00%)	17 (65.38%)	
III	16 (17.39%)	0 (0.00%)	1 (4.00%)	7 (28.00%)	8 (30.77%)	
0	12 (13.04%)	12 (75.00%)	0 (0.00%)	0 (0.00%)	0 (0.00%)	
Tumor lymphocyte infiltration						<0.001
0	52 (56.52%)	16 (100.00%)	23 (92.00%)	13 (52.00%)	0 (0.00%)	
10.0–59.0	26 (28.26%)	0 (0.00%)	2 (8.00%)	8 (32.00%)	16 (61.54%)	
≤10%	11 (11.96%)	0 (0.00%)	0 (0.00%)	3 (12.00%)	8 (30.77%)	
>59%	3 (3.26%)	0 (0.00%)	0 (0.00%)	1 (4.00%)	2 (7.69%)	

^*a*^ *n* (%).

^*b*^ Fisher's exact test for count data with simulated *P*-value (based on 2,000 replicates).

We performed 5R 16S rRNA analysis to validate the presence of bacterial populations in both neoplastic and corresponding non-neoplastic tissue samples. After eliminating bacterial contamination in the negative controls and excluding samples of low quality, we successfully identified a total of 1,574 species at the taxonomic level of species, 556 at the genus level, 159 at the family level, 74 at the order level, 41 at the class level, 23 at the phylum level, and finally 1 domain.

**Fig 2:**
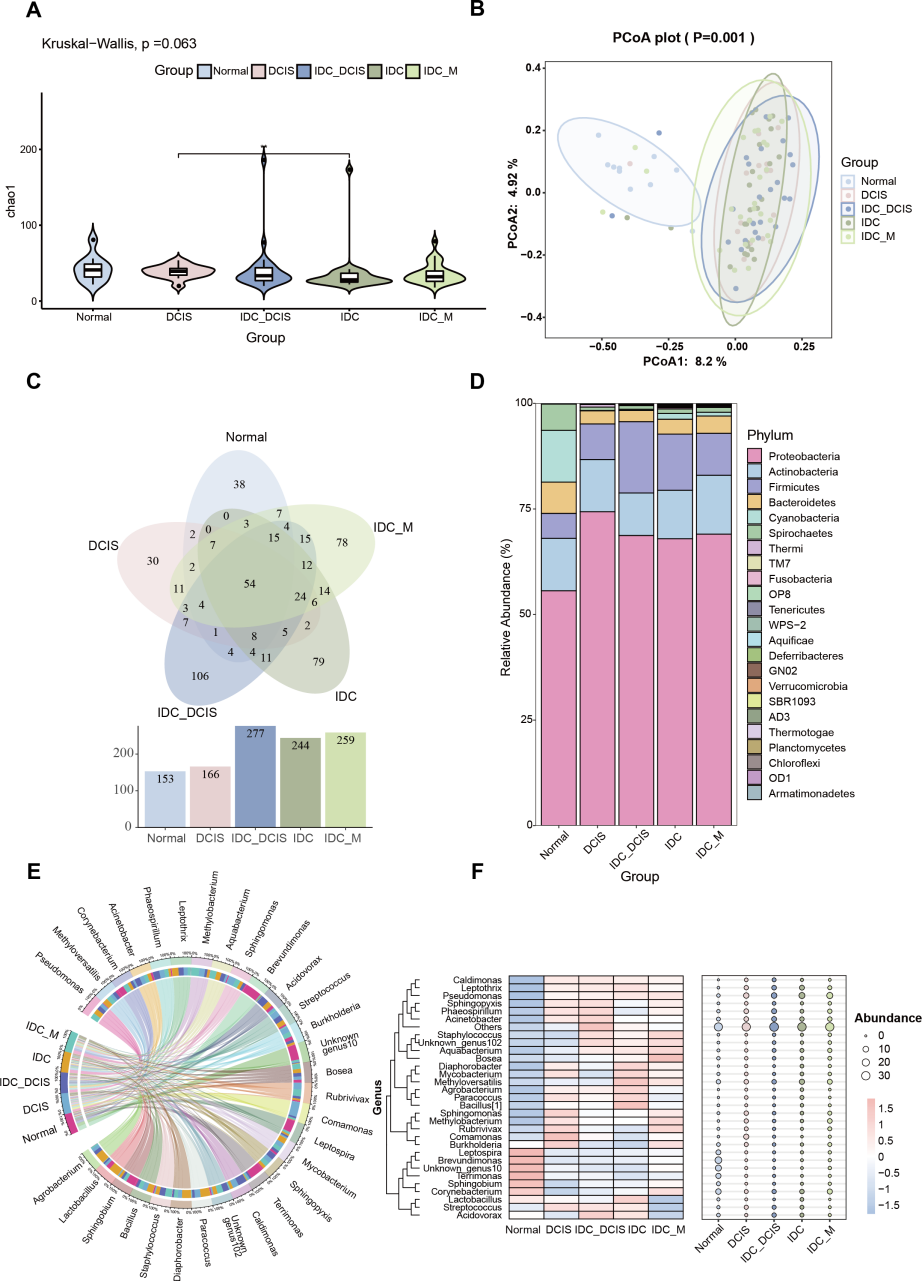
Analysis of the bacterial microbiota composition in adjacent healthy tissues and breast cancer tissues. (A) The α diversity index of the microbiota in neighboring healthy tissues and different stages of breast cancer; (B) β-diversity index indicating the variation in microbiota composition between adjacent healthy tissues and ductal breast cancer; (C) investigation of the correlation between tumor and microbiota in the adjacent non-cancerous tissue at the taxonomic level of genus; (D) proportional bar charts illustrating the Phylum-level characteristics of intratumoral microbial composition across all experimental groups. (E) The relative abundances of the top 30 most prevalent microorganisms across different categories. (F) The heat map with bubbles visually depicts the spatial distribution of microorganisms across different groups, where the size of each bubble corresponds to the relative abundance of microorganisms. Bubbles in red indicate a higher expression level, while those in blue suggest a lower expression level. (Normal: paracancer tissue; DCIS: ductal carcinoma *in situ*; IDC_DCIS: infiltrating ductal carcinoma with ductal carcinoma *in situ*; IDC: infiltrating ductal carcinoma; IDC_M: infiltrating ductal carcinoma with distant metastasis)

To evaluate disparities in taxonomic composition and microbial diversity between tumor and normal tissues, we conducted comprehensive analyses on both alpha and beta diversities. No significant variations in richness and evenness levels were observed among adjacent tissues or all collected tumor tissues, as evidenced by the Chao1 index (*P* = 0.063; *P* = 0.098, Fig. 2A; Fig. S1A). An analysis utilizing PCoA with Bray-Curtis distance measures demonstrated clear segregation between tumor and non-tumor tissue clusters. Additionally, the microbial community composition in tumor tissues exhibited significant differences from that of the corresponding adjacent tissues (*P* = 0.001, Fig. 2B; Fig. S1B).

The adjacent normal group and paired tumor group exhibited a shared total of 115 microbial consortia at the genus level. Furthermore, it was found that the microbial abundance and diversity within BC tumor tissues were markedly higher than those observed in the surrounding normal tissues (Fig. S1C). Employing a Venn diagram to analyze the microbial profile in tumor tissue, we observed a gradual increase in microbial abundance during the progression from ductal carcinoma *in situ* to infiltrating ductal carcinoma with concurrent presence of ductal carcinoma *in situ*, ultimately culminating in infiltrating ductal carcinoma with distant metastasis (Fig. 2C). The distinct microbial population present within the tumor may be strongly associated with the disease status of breast cancer. The predominant microorganisms identified included *p_Firmicutes*, *p_Actinobacteria*, *p_Proteobacteria*, *p_Cyanobacteria*, *p_Bacteroidetes,* and *p_Spirochaetes*(Fig. 2D). At the genus level, the proportional representation of *g_Pseudomonas* were 10.34% in DCIS, 9.34% in IDC_DCIS, 12.83% in IDC, and 13.96% in IDC_M. Notably, both *g_Pseudomonas* and *g_Corynebacterium* exhibited a gradual increase (Fig. S1D). Importantly, the microbial spectrum undergoes dynamic changes throughout the progression and evolution of the disease. Additionally, there exists microbial composition variation within tumors among patients with breast ductal carcinoma at the same disease stage, thereby indicating individual heterogeneity (Fig. S1E). We conducted further investigations into the intratumor microbiome in breast cancer tissues by employing species relationship Circos, as well as utilizing a combination of differential heat map and bubble map analysis (Fig. 2E and F).

### Association between tumor-resident *Pseudoalteromonas elyakovii*, tumor tissue, and para-carcinoma tissue in patients with BC

**Fig 3:**
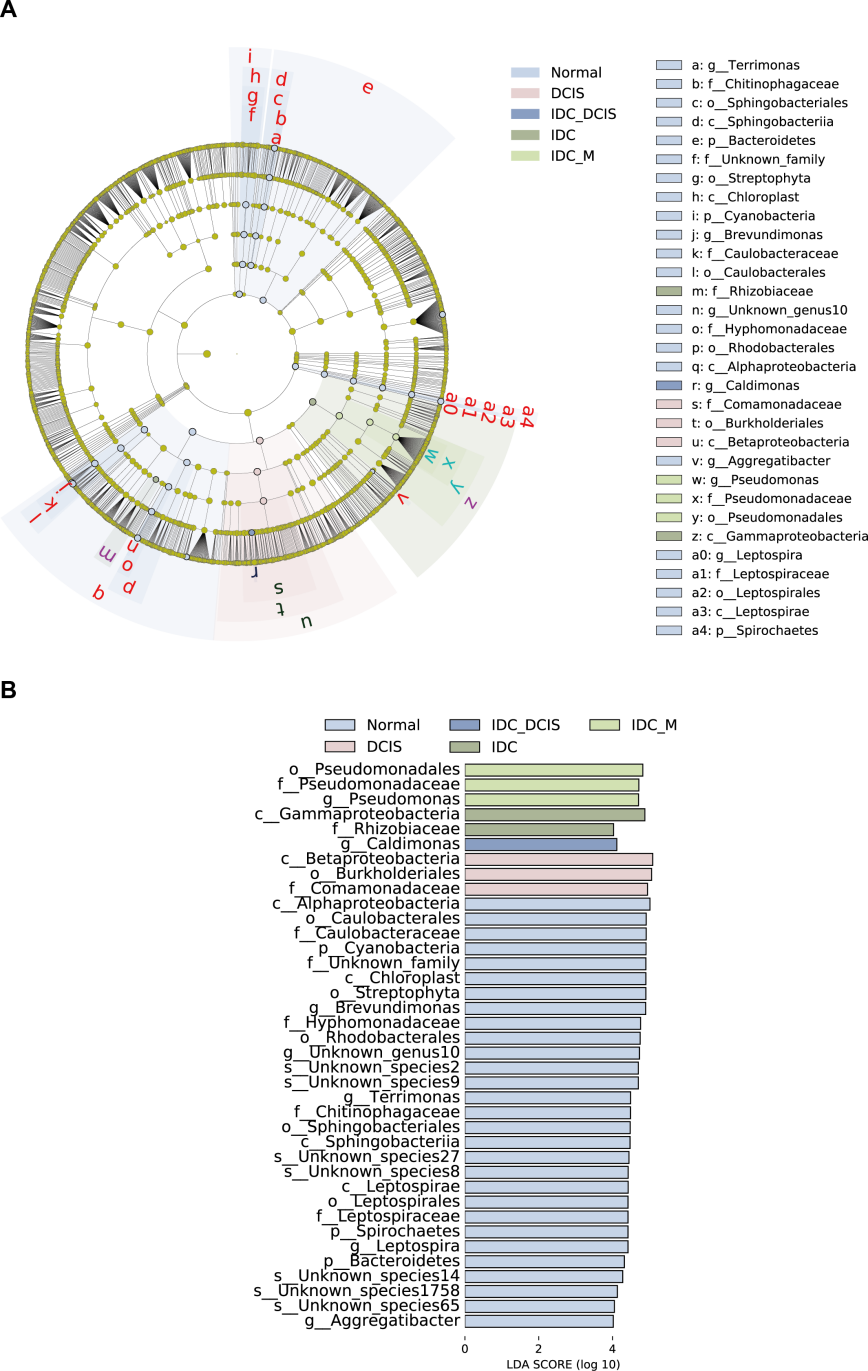
The distinctive features of the microbiota across the two groups were delineated utilizing LEfSe and LDA analyses. (A) The hierarchical taxonomy of the detected microbial community is represented by a Cladogram generated through LEfSe. Red nodes and blue nodes, respectively, stand for species with relatively high abundance that show significant difference between the tumor and normal groups. (B) The histogram displaying LDA scores revealed substantial differences in microbial species and their prevalence between tumor and normal tissue groups (LDA refers to linear discriminant analysis, while LEfSe denotes linear discriminant analysis effect size).

To delve deeper into these results, we performed high-dimensional category comparisons (LDA score > threshold of 4) using LEfSe based on Wilcoxon or Kruskal-Wallis rank sum tests. This analysis unveiled significant disparities in the prevalence of intratumoral microbial communities between normal and tumor tissues (Fig. 3A and B). The predominant bacterial taxa identified in tumor tissues included *p_Proteobacteria*, *g__Pseudomonas*, *g__Phaeospirillum*, *s__Phaeospirillum fulvum*, and *s__Pseudoalteromonas elyakovii*.

**Fig 4:**
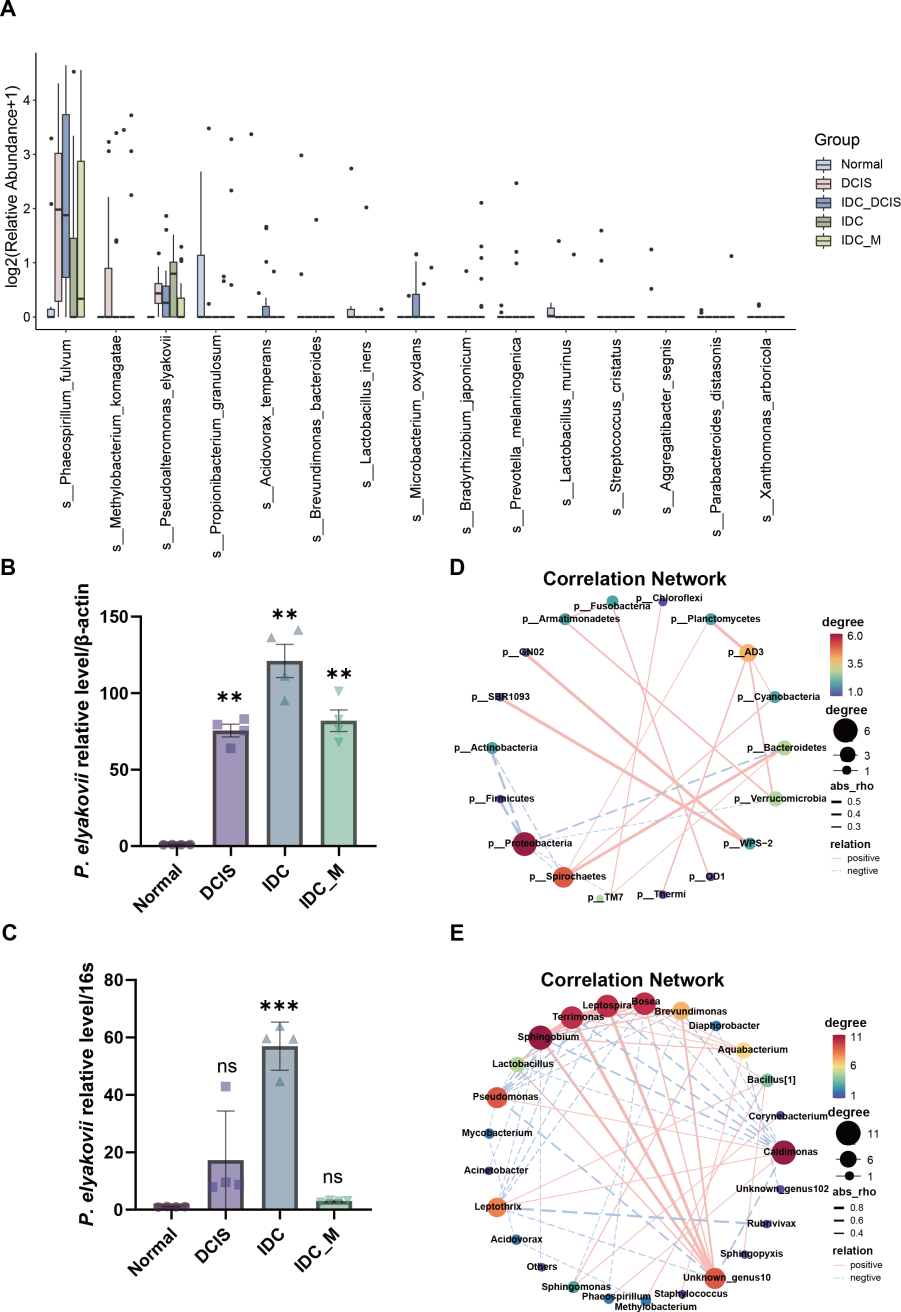
The analysis of microbial variation between tumor and neighboring tissues aimed to identify distinct flora with significant differences. (A) The levels of differentially expressed bacteria at the species level in various neoplastic and adjacent benign tissues using the Kruskal-Wallis test with biological duplicates. The threshold for statistical significance was set at *P* < 0.05. (B, C) qRT-PCR was employed to confirm the varying levels of *Pseudoalteromonas elyakovii* in freshly sterilized tissues. (D, E) A correlation network was employed to examine the connections among microorganisms at both the phylum and genus levels.

Building upon the aforementioned findings, we conducted a comprehensive analysis of the distinct microbial communities residing within tumors at the genus level, comparing various groups in both the adjacent non-cancerous and tumor cohorts. The predominant bacterial species identified in the breast cancer tumor group included *g__Pseudomonas*, *g__Phaeospirillum*, *g__Agrobacterium,* and *g__Pseudoalteromonas* (Fig. S2A). The prevalence of *Pseudoalteromonas elyakovii* was found to be significantly higher within tumor tissues as opposed to their normal tissue counterparts, exhibiting a gradual increase throughout the progression of the disease (Fig. 4A). To demonstrate this phenomenon, we obtained fresh human breast cancer samples meeting the specified criteria from the surgical department and subsequently confirmed the presence of *P. elyakovii* in both tumor and non-tumor tissues using qRT-PCR analysis. The results revealed a significant increase in the abundance of *P. elyakovii* within the tumor group compared to the adjacent normal tissues, with a gradual elevation observed as the disease progressed. Interestingly, patients diagnosed with infiltrating ductal carcinoma and distant metastasis from the primary tumor site exhibited a noteworthy decrease in *P. elyakovii* abundance; however, it remained higher than that found in neighboring tissues. These findings are consistent with those obtained through 5R 16S rRNA sequencing analysis (Fig. 4B and C).

Additionally, we investigated the correlation between taxa present in the tissues. According to the Spearman correlation analysis, we observed the intricate network structure of “Phylum” and “Genus.” At the phylum level, a positive association was observed between *p__Bacteroidetes* and the abundance of *p__Spirochaetes* and *p__TM7*, while *p__Proteobacteria* exhibited a negative correlation with both *p__Spirochaetes* and *p__Verrucomicrobia* (Fig. 4D). These findings suggest potential interactions among microbial communities that may influence the complex tumor microenvironment (TME) in vivo, thereby potentially playing a role in the initiation and advancement of pathological conditions. The influence of the microbial consortium on the overall body can be visualized by examining the intricate network of microbial interactions within the tumor even when considering their relationships at the genus level (Fig. 4E).

### Functional prediction suggests that intratumor microbes may be involved in ductal carcinoma progression

**Fig 5:**
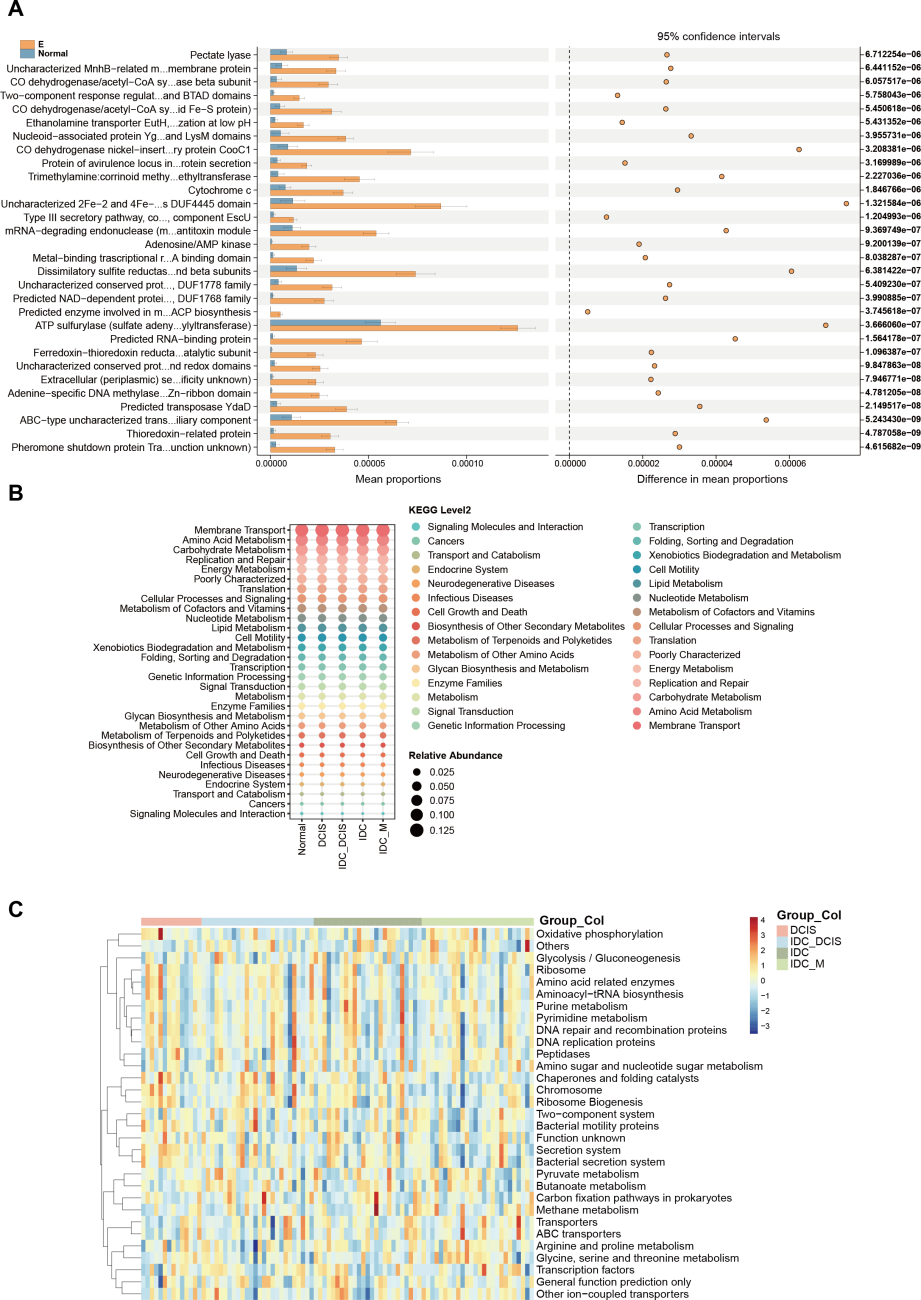
Functional profiling prediction of bacterial microbiota was conducted. (A) The COG database, in collaboration with PICRUSt2, was utilized to forecast the roles of bacterial microbiota within tissue samples. The KEGG database, in conjunction with PICRUSt2, was applied to forecast the functional capabilities of bacterial microbiota within tissues. Subsequently, the findings from the KEGG pathway analysis were detailed at two distinct levels: Level 2 (B) and Level 3 (C).

Based on the analysis of 16S amplicon sequencing data and comparison with the COG database, we identified and categorized relevant genes and pathways based on their functional relationships (Fig. 5A). The microbiome within tumor tissues exhibited a significant upregulation of ABC-type uncharacterized transport system, ATPase component compared to normal tissues.

Similarly, by integrating the 16S sequencing data in combination with the eggNOG and KEGG databases, we performed a comprehensive analysis on the functionalities of the top 30 prevalent microbiota. The functions of these microbiota primarily encompassed Membrane Transport, Replication and Repair, Translation, Cancers and Amino Acid Metabolism, Carbohydrate Metabolism, Energy Metabolism, as well as Poorly Characterized categories. By integrating the 3rd Level data obtained from KEGG, it becomes evident that microorganisms present in tumors exert influence on diverse biological processes, including DNA repair and recombination proteins, ABC transporters, Transporters, Two-component system, Purine metabolism, Bacterial motility proteins, and Pyruvate metabolism (Fig. 5B and C).

### *Pseudoalteromonas elyakovii* promote breast cancer cell migration and proliferation

**Fig 6:**
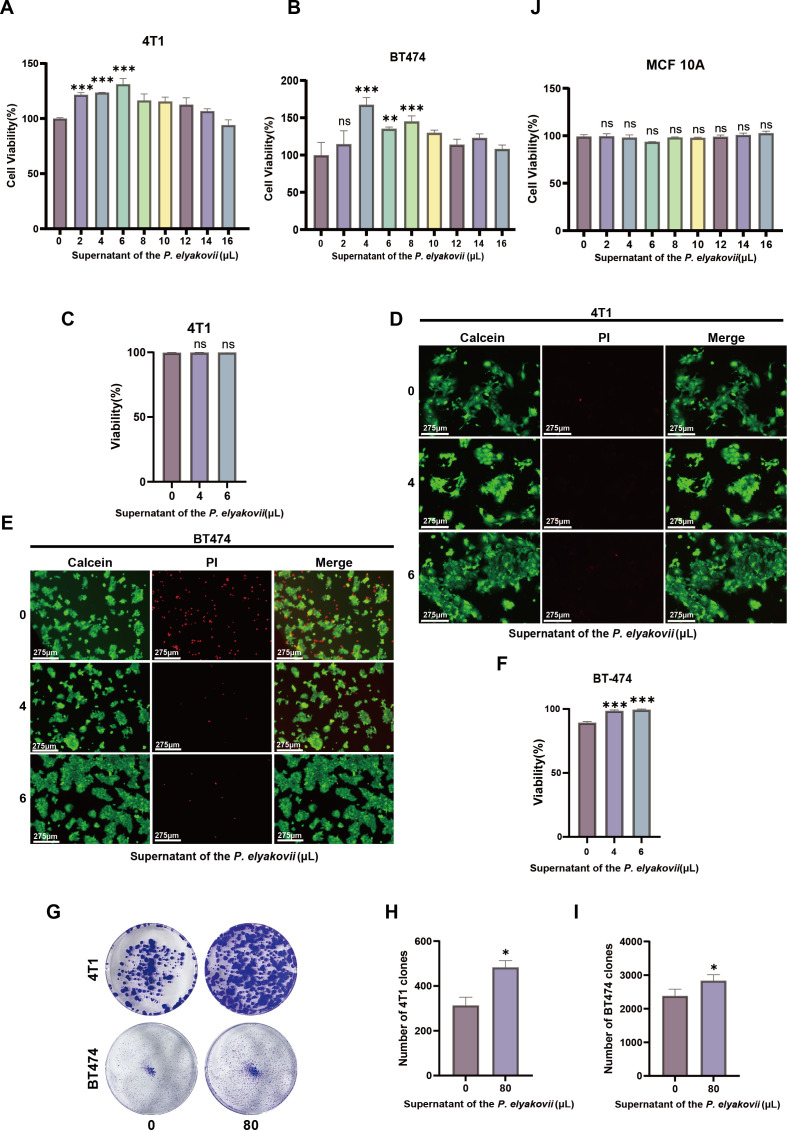
Breast tumor cell proliferation is promoted by the supernatant of *P. elyakovii*. (A) The activity of 4T1 in the supernatant of *P. elyakovii* was evaluated using the CCK-8 assay for assessment purposes. (B) The impact of exposing BT474 cells to *P. elyakovii* supernatant was assessed using the CCK-8 method which was employed to assess cellular viability. (C, D) Representative visuals and quantification of Live-Dead Cell Staining experiments conducted on 4T1 cells following exposure to varying concentrations of supernatant; Scale bar, 275 µm. (E, F) Representative visuals and quantification of Live-Dead Cell Staining experiments conducted on BT474 cells following exposure to varying concentrations of supernatant; Scale bar, 275 µm. (G–I) 4T1 cells and BT474 cells were cultured in 6-well plates and treated with supernatant from *P. elyakovii*. The count of communities was performed on day 14 post-inoculation. (J) The impact of the supernatant from *P. elyakovii* on MCF10A cell viability was assessed using the CCK-8 assay. The data are presented as the mean ± SEM, with a sample size of *n* = 3. *P < 0.05, **P < 0.01, and ***P < 0.001.

To explore the roles carried out by these bacterial species, it was hypothesized that the liquid fraction of the bacterial solution contained macroscopic molecules and metabolites synthesized by the bacteria. In our study, we employed breast cancer cell lines and normal mammary epithelial cell lines to assess the impact of *P. elyakovii* metabolites on cell migration using Cell Counting Kit-8 assay (CCK8), Transwell migratory assay, and Wound healing assay. In view of the limited existing literature on the involvement of *P. elyakovii* in breast cancer cell progression, we conducted comprehensive *in vitro* and *in vivo* experiments employing *P. elyakovii* obtained from Mingzhoubio (Ningbo) as a positive control. The CCK8 assay results demonstrated a dose-dependent stimulation of cancer cell growth within a specific range by the filtered supernatant of *P. elyakovii* solution, devoid of viable bacteria. Furthermore, an enhanced proliferative effect was observed with increasing dosage (Fig. 6A and B). Notably, the supernatant from *P. elyakovii* did not significantly influence the proliferation of normal mammary epithelial cells (Fig. 6J). We conducted additional experiments to evaluate the viability of cancer cells treated with *P. elyakovii* supernatant by employing Calcein AM and PI staining. The results demonstrated that the treatment induced a proportional increase in membrane permeability. The red staining of deceased cells demonstrated a decrease as the quantity of supernatant increased. Concurrently, there was an enhancement in the green fluorescence signal corresponding to the supernatant, resulting in a reduction in the mortality rate of viable cells. The qualitative assessment of fluorescence was performed using fluorescence imaging (Fig. 6D and E), while the green and red fluorescence intensities were quantitatively determined (Fig. 6C and F). Compared with the control group, the supernatant of *P. elyakovii* also demonstrated a stimulatory effect on colony formation in 4T1 and BT474 cells (Fig. 6G through I).

**Fig 7:**
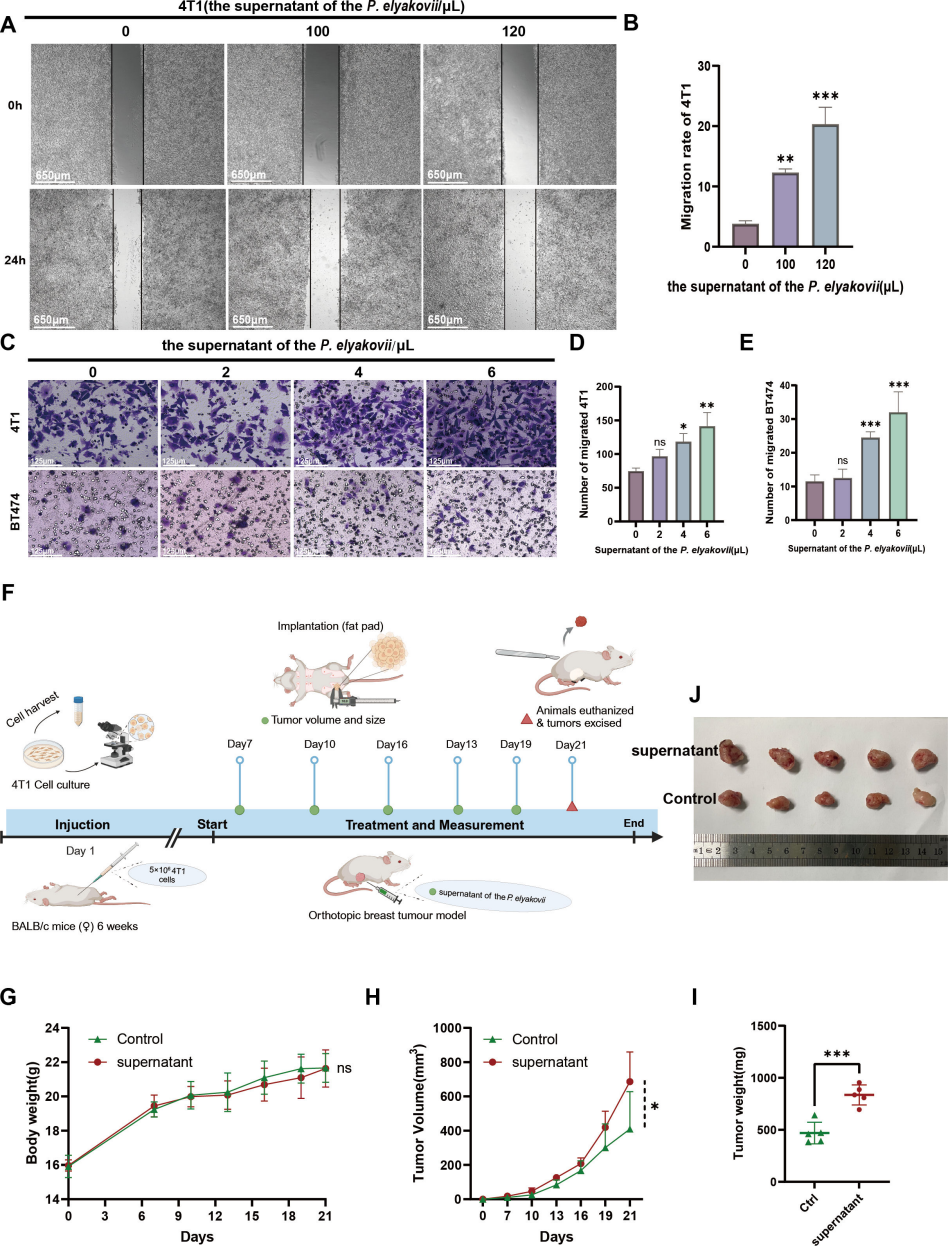
The migration of tumor cells was facilitated by the supernatant derived from *P. elyakovii*. (A, B) Visual representations and measurement of 4T1 cell scratch assay following exposure to tumor cell supernatant; Scale bar, 650 µm. (C–E) The impact of *P. elyakovii* supernatant on the movement of 4T1 and BT474 cells was assessed using the transwell assay for cell migration; Scale bar, 125 µm. Results are depicted as the mean ± SEM, with *n* representing the sample size of 3. Significance is denoted by * for *P* < 0.05, ** for *P* < 0.01, and *** for *P* < 0.001. (F) The following describes the workflow for the treatment of BALB/c mice with *P. elyakovii* supernatant. (G) The weight of each mouse was recorded at 3-day intervals for a period of 21 days. (H) Tumor volumes were quantified at 3-day intervals. (I) The precise documentation of the weight of the dissected tumors. (J) Representative images of 4T1 tumors from various experimental groups.

Upon introduction of *P. elyakovii* supernatant, a significant enhancement in the percentage of cell scratch healing was observed in 4T1 cells compared to control samples (Fig. 7A and B), and this rate of scratch healing exhibited an increasing trend with escalating concentrations of bacterial supernatant. Furthermore, the transwell assay results revealed a significant increase in cell density per unit area when examining 4T1 cells under an electron microscope following the introduction of *P. elyakovii* supernatant, indicating an enhanced migratory capacity of the cells. A similar trend was observed in the BT474 cell line (Fig. 7C through E). Collectively, these findings suggest that the culture medium of *P. elyakovii* facilitated the proliferation and motility of diverse breast cancer cell variants, potentially attributed to the presence of bioactive components in the metabolites derived from *P. elyakovii*.

The 4T1 cell line was employed in a 6-week-old BALB/c mouse model (Fig. 7F and J). Tumor cells were transplanted *in situ* into the mammary gland of mice as a control group in addition to another group that was additionally treated with the supernatant of *P. elyakovii*. As illustrated in Fig. 7G, the supernatant did not exert a notable influence on the body weight of BALB/c mice. As observed previously, treatment of *P. elyakovii* supernatant resulted in a dose-dependent promotion of tumor development *in vivo* (Fig. 7H and I). The results of this study indicate that the supernatant of *P. elyakovii* has the capacity to stimulate the proliferation of BC cells *in vivo*.

### scRNA-seq elucidated the distribution pattern of immune cells at different stages of breast ductal carcinoma development

**Fig 8:**
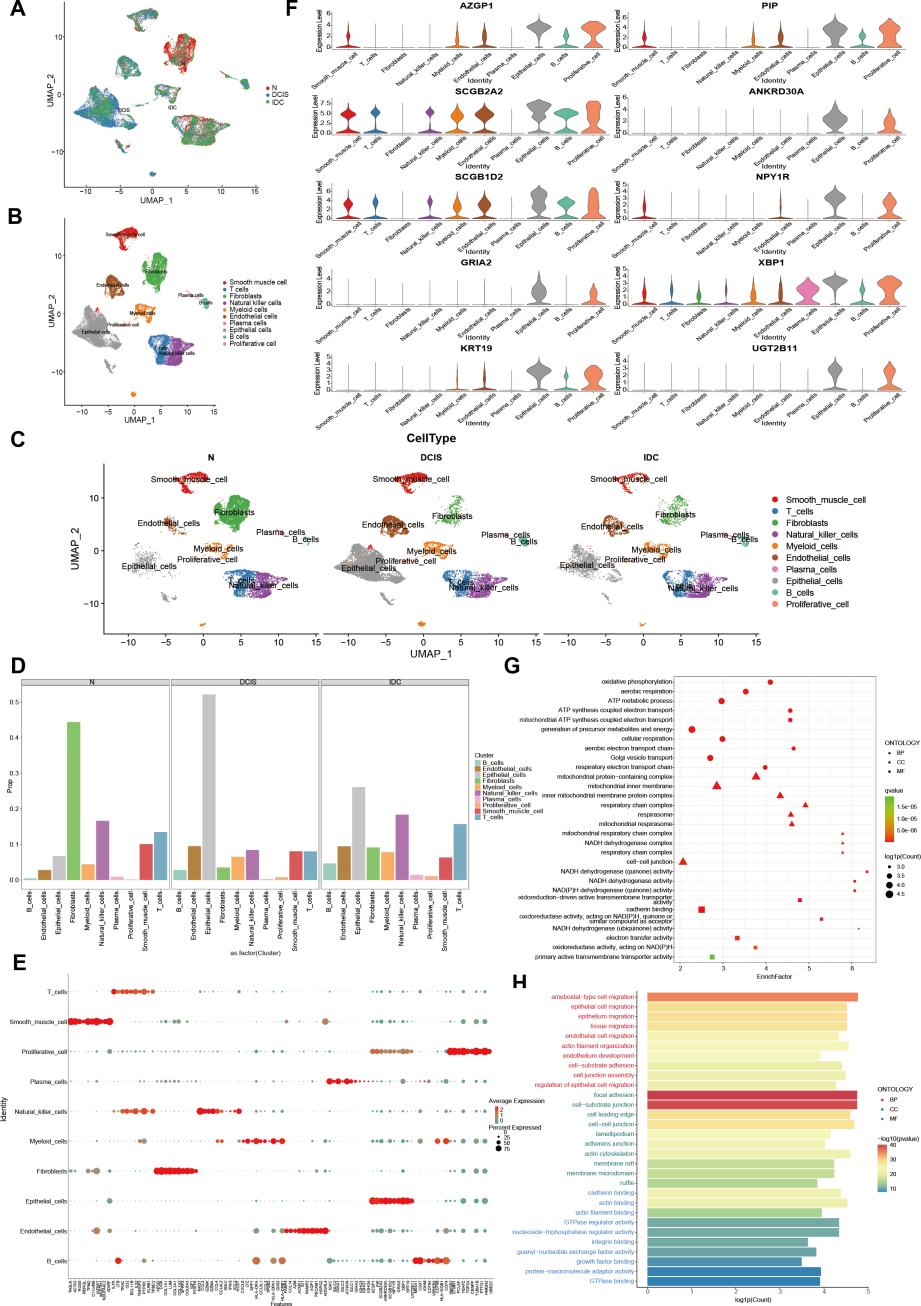
A scRNA-seq survey of cells at different stages of development within the mammary gland ducts. (A) UMAP plot of 26,684 cells from three patients diagnosed with ductal carcinoma of the breast, as part of the study in question, demonstrates cell origin via coloration. (B, C) UMAP plot showing cell clusters including smooth muscle cell, T cells, fibroblasts, myeloid cells, epithelial cells, natural killer cells, endothelial cells, plasma cells, B cells, and proliferative cells. (D) The percentage of cells belonging to each cell type within a given sample is indicated. (E) The dot plot illustrates the expression of DEG marker genes across various samples. (F) The stacked violin plot illustrates the expression levels of selected marker genes in epithelial cell clusters. (G, H) The top GO terms were found to be enriched in epithelial and endothelial cells. The degree of intensity represents the adjusted *P*-value of each hallmark. The size of the dot indicates the number of genes associated with each hallmark. The Wilcoxon signed-rank test was employed for the purpose of assessing the difference. (N: Paracancer tissue, DCIS: ductal carcinoma *in situ*, IDC: Infiltrating ductal carcinoma).

In combination with other reports, the existence of microorganisms in breast tissue was verified through 5R 16S rRNA sequencing and qRT-PCR experiments. However, it fails to fully depict the alterations in microbial distribution across various cell types. Therefore, in order to clarify the intratumoral microbial single-cell landscape of breast ductal carcinomas with different histological grades, we analyzed BC scRNA-seq data from three tissue samples (for detailed clinical and pathological information about the patients, Table S3) of BC patients. A total of 26,684 cells were generated for subsequent clustering analysis (Fig. 8A). When the cells were projected onto a 2D Uniform Manifold Approximation and Projection plot, the cells were categorized into a total of 10 distinct cell clusters. By reviewing the marker genes, these 10 cell categories were annotated as follows: smooth muscle cells, T cells, natural killer cells, fibroblasts, endothelial cells, myeloid cells, plasma cells, epithelial cells, B cells, and proliferative cells (23–26) (Fig. 8B and C). To reveal the heterogeneity of cellular profiles at different stages of breast cancer disease, key cellular annotation groups were compared between different stages of breast cancer and paracancer tissue. In particular, epithelial cells, endothelial cells, and B cells were enriched mainly in DCIS and IDC breast cancers but were rarely expressed in normal tissues; in contrast, fibroblasts showed high expression in normal tissues adjacent to breast cancers (Fig. 8D; Fig. S2A, Table S4 and S5). Expression analysis of marker genes commonly employed to label-specific cellular annotation populations revealed that numerous genes demonstrate limited specificity or extremely low levels of expression (Fig. 8E). As the cell cluster with the most pronounced difference in proportions between tumor and paracancer tissue, epithelial cells were further analyzed for uniquely characterized genes in each cell annotation cluster (Fig. 8F). Oxidative phosphorylation, generation of precursor metabolites, and energy were observed in epithelial cells, B cells, endothelial cells, and other cell clusters. The expression of important signaling pathways such as immune response-regulating signaling pathway, secretory granule membrane, ameboidal-type cell migration, and sulfur metabolism was increased (Fig. 8G and H; Fig. S2B and C, Table S6 and S7).

### scRNA-seq revealed enrichment of microbes in immune cells

**Fig 9:**
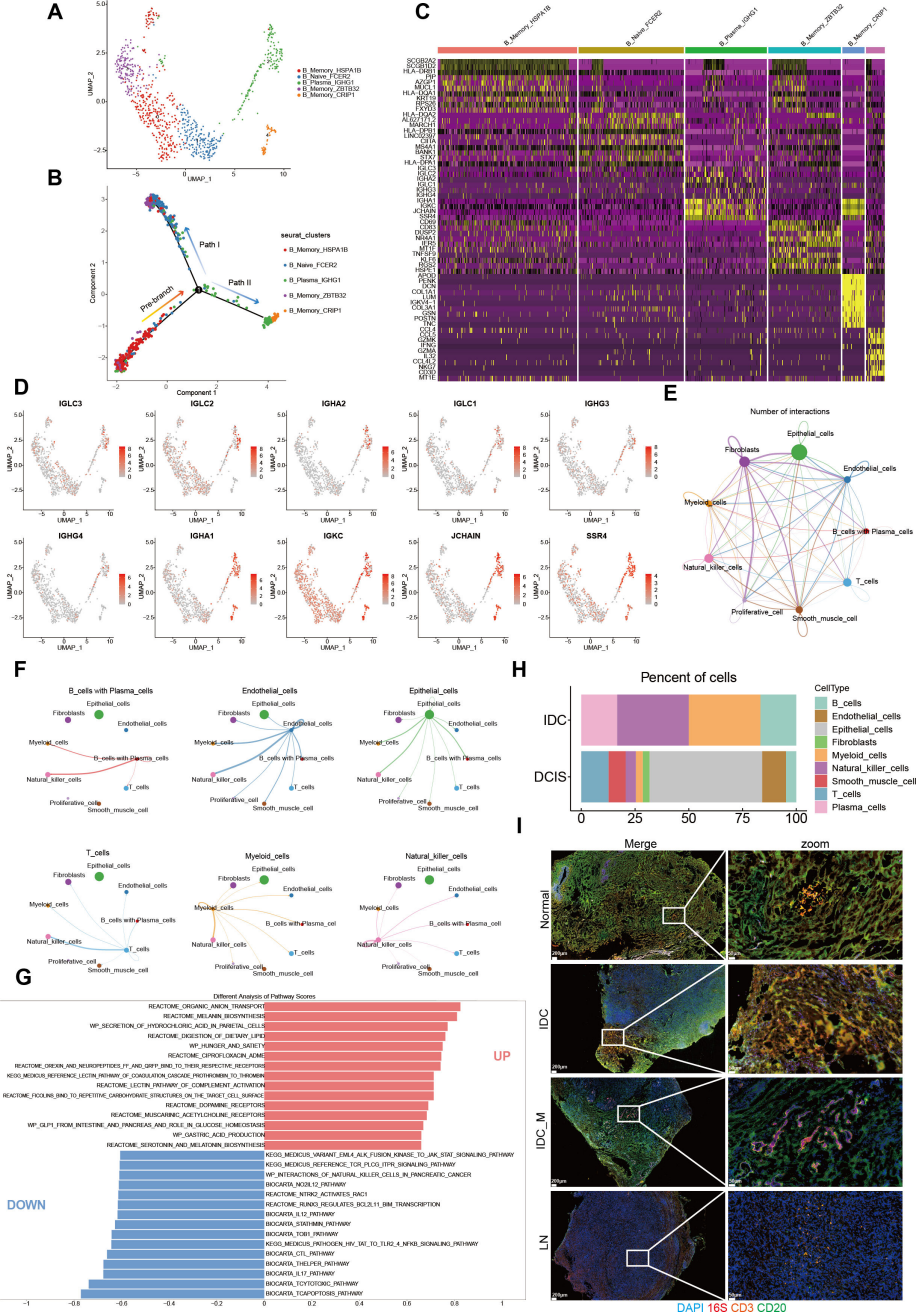
The tumor-associated microbiota facilitates the recruitment of immune cells and enhances the immune response against tumors. (A) UMAP plot depicting five distinct B cell clusters. (B) Pseudotime trajectory analysis of B cells and the mapping of B cell subpopulation distributions. (C) Heatmap of the first 60 DEGs in each B cell cluster. (D) The UMAP plot presenting the expression of chosen marker genes on 5 B cell clusters. (E, F) Intercellular interactions. Arrows and edge colors point out the direction. The size of the circle is in proportion to the quantity of cells in each cell group. The thickness of the edge indicates the number (E) and the intensity (F) of the interactions between populations. (G) Bar plot of GSVA analysis comparing specifically enriched pathways in three samples of ductal carcinoma of the breast. (H) Proportion of microbial abundance in each cell type in each sample. (I) Representative images of double immunofluorescence (IF) staining combined with SweAMI probe *in situ* hybridization, were obtained from four samples representing Paracancer tissue (Normal), invasive carcinoma without lymph node metastasis (IDC), invasive carcinoma with lymph node metastasis (IDC_M), and distant metastatic lymph nodes (LN). Scale bar, 200 µm.

Unlike traditional scRNA-seq data analysis, we found that microbial RNAs are present not only in parenchymal cells but also in a wide range of immune cells by analyzing the number of microbial reads in the initial matrix. The microbial abundance in each cell of the sample was calculated through scRNA-seq analysis. The results indicated that no substantial evidence of microbial residency in immune cells was found in paracancerous tissues. Nevertheless, the residency of microbes of varying abundance in immune cells was noticed in tumor samples(Fig. 9H; Table S12). The predominant cell type in tumor tissue is the parenchymal cell, mainly composed of epithelial cells and myeloid cells. Thus, most of the microorganisms in the tumor are enriched in these cell types. Among them, in addition to parenchymal cell microbes, in DCIS samples, T cells and B cells were more enriched. In IDC samples, microorganisms were most prevalent in myeloid cells and natural killer cells.

In this study, B cells, which exhibit the most significant differences in proportions between different stages of breast ductal carcinoma and normal tissue, were further classified into five subpopulations, each possessing its own unique characteristic genes (Fig. 9A, C and D; Fig. S2D and E).

To ascertain the developmental pathway of B cell clusters, pseudotime analysis was conducted. Pseudotemporal trajectories of B cell clusters maintain two differentiation pathways, commencing with naive B cells and B_Memory_HSPA1B and culminating in a continuous transition to memory B cells at one extremity or plasma cells and plasmablasts at the other extremity (Fig. 9B; Table S8). Interestingly, the pattern of differentiation varies among different stages of ductal carcinoma. B cells in Normal and DCIS tend to differentiate from pre-branching to pathway II and less frequently cluster in pathway I. In contrast, B cells in IDC are more likely to cluster pre-branching and differentiate into pathway I rather than pathway II (Fig. S2F). Overall, these findings imply that different stages of ductal carcinoma of the breast also present distinct patterns of differentiation under the same histological typing. Specifically, B cells in IDC tend to differentiate into memory cells and plasma cells, which play a role in humoral immunity. Nevertheless, B cells in DCIS are more prone to aggregate and function as Naive cells.

To identify potential interactions among different cell clusters in ductal carcinoma of the breast within the tumor microenvironment (TME), we carried out CellChat analyses to assess intercellular signaling links (27). Although the number of interactions and the intensity of the interactions among the major cell types were relatively similar in the different samples, significant differences still existed (Fig. S2G). Analysis reveals that epithelial cells have more interactions with other cell clusters (Fig. 9E; Table S9), also demonstrated a stronger interaction with immune cells such as B cells, plasma cells, and natural killer cells (Fig. 9F; Table S10). Gene set variation analysis (GSVA) was also carried out to explore the B cell subpopulations that mediate potential differential pathways. GSVA results indicated that reactome organic anion transport was significantly up-regulated in ductal carcinoma, facilitating the delivery of biologically active secondary metabolites secreted by the intratumoral flora to the receptor and influencing the dynamic and complex environment of the TME. The secretion of hydrochloric acid in parietal cells, which prompts the cells to secrete hyaluronic acid, is a molecule secreted by cancer-associated fibroblasts (CAFs), and it exerts a substantial influence within the tumor microenvironment. In addition, other enrichment pathways, such as reactome melanin biosynthesis and reactome digestion of dietary lipid, were also significantly up-regulated (Fig. 9G; Table S11).

### Double immunofluorescence staining combined with SweAMI probe *in situ* hybridization

Current investigations have revealed the role of the gut microbiome composition possesses the potential to modulate the tumor immune microenvironment by regulating targeted infiltration of immune cells (TIME) (12, 28–31). We propose that the microbiota residing within tumors serves a comparable function. By employing a combination of double immunofluorescence staining and SweAMI probe *in situ* hybridization, we conducted an investigation into the infiltration of immune cells within the tumor microenvironment. The immunofluorescence analysis was performed to investigate the presence of intratumoral microbes and the expression of CD3- and CD20-related organisms in four breast cancer samples, including adjacent tissue, non-metastatic invasive cancer, metastatic invasive cancer with nodal involvement, and distant metastatic lymph nodes. The results revealed inter-individual and regional variations in the microbial community. In general, the presence of microorganisms was higher in invasive cancer cases without lymph node metastasis and those with lymph node metastasis compared to adjacent tissues. Furthermore, a significant number of CD3 cells and CD20 cells were observed in close proximity to the intratumoral microorganisms. These findings suggest that the intratumoral flora may play a pivotal role in modulating immune cell activity (Fig. 9I).

## DISCUSSION

Based on previous reports, we postulated that the tumor microbiota might promote or inhibit cancer progression and metastasis through diverse mechanisms, such as modulating local and systemic immune responses, altering the tumor microenvironment, and inducing genomic instability (9, 12, 32). This study probed into the composition and diversity of the intratumoral microbiota in untreated patients with the first diagnosis of BC. We conducted a comprehensive and integrated analysis and identified different abundances of microbiota within the tumors and adjacent normal tissues of BC patients. This view is underpinned by the accumulating evidence from multiple recent comprehensive analyses of the intratumoral microbiota, which report a close connection between microbiota and TME (33–35). The α-diversity of the collected tumor tissues did not show a significant difference from that of the matched adjacent normal tissues. Moreover, the β-diversity of the collected tumor tissues, as revealed by PCoA of Bray-Curtis distance, indicated that the microbial community composition and diversity of the tumor tissues differed significantly from those of the adjacent normal tissues when compared with those of the non-tumor tissues, presenting unique tumor microbiota characteristics.

5R 16S rRNA disclosed that the majority of microorganisms were expressed at a higher abundance in BC tissues compared to adjacent normal tissues. Among these differences, *P. elyakovii* exhibited aberrant expression abundance in tumor groups compared with adjacent normal tissue, and the abundance gradually rose during disease progression. The genus *Pseudoalteromonas* was described by Gauthier et al. (36). *Pseudoalteromonas* is a major branch of the marine biota belonging to the order γ-*Anamorphobacteria*, which is under the category of the order *Alternaria* and inhabits all known non-geothermal marine communities.

*P. elyakovii*, a bacterium that generates extracellular polysaccharides, might also produce secondary metabolites with biological activity. Therefore, we propose that the aberrant expression of microbial abundance, mainly *P. elyakovii*, is a crucial predictor of disease progression and survival prognosis in BC patients, providing novel insights for the future clinical applications of the intra-tumor microbiota. These findings imply that *P. elyakovii* holds potential for applications in biotechnology and biomedical research. It has also been shown that a large number of the bacteria in tumor tissue are located intracellularly and that they exist in cancer cells and immune cells (12). Coinciding with the scRNA-seq results we monitored, this indicates that there seems to be no distance barrier between the microbes and the tumor cells, suggesting that there are direct or indirect interactions between them. On this basis, we postulated that the metabolites of this microorganism might serve as unique constituents of the tumor microenvironment, which could enable the highly abundant expression of these bacterial metabolites to create favorable ecological niches for tumor cells and exert an influence on them. Complex crosstalk between the host and microbiota within the TME influences tumor progression by regulating oncogenic pathways, modulating immune responses, and interacting with microbiota-derived metabolites (37–39).

Changes in microbial abundance are considered to be the process of microbial interaction with human host cells. *In vitro* experiments have demonstrated that the supernatant of *P. elyakovii* promotes the proliferation of tumor cells within a certain range of small doses, and that the pro-tumorigenic effect of tumor cells is inhibited when the dose exceeds a certain threshold. Transwell analysis revealed that the addition of the appropriate volume of *P. elyakovii* supernatant led to a significant increase in the number of cells per unit area under the electron microscope, indicating enhanced proliferation and migration of the cells. The relationship between bacterial metabolites and tumors was investigated through a mouse breast cancer *in situ* tumor model. *In vitro* experiments revealed that the supernatant of *P. elyakovii* exerted a significant pro-growth effect on transplanted tumors compared with the controls, indicating the existence of potent components of *P. elyakovii* metabolites that facilitate tumor progression. Preliminary exploration of the interaction between local tumors and intratumoral microbiota and their role in remodeling TME may assist in understanding the mechanisms by which microorganisms act locally and facilitate research on new therapeutic strategies for BC. This study discloses the initial role relationship and innovative findings between *P. elyakovii* and ductal carcinoma in the breast.

In conclusion, our study reveals the existence of an intratumoral microbiota specific to patients with BC. The enrichment of *P. elyakovii* features might accelerate the disease progression among patients having ductal carcinoma of the breast. Intratumor microbiota reshape the tumor microenvironment, thereby influencing the evolution of local tumor cells, which suggests the potential clinical significance of intratumor microbiota for cancer immunotherapy. Detailed information regarding the specific components and bioactivities in the supernatant of *P. elyakovii* remains limited. However, the molecular mechanisms regarding precisely how they interact with tumor cells still need to be explored in depth to clarify the detailed mechanisms associated with the altered microbiota residing in tumors and their dynamics.

## MATERIALS AND METHODS

### Patient recruitment

Between 23 March 2023 and 4 June 2024, a total of 91 patients were enrolled in this study.

### Cell lines and cell culture

The cell lines utilized in this investigation were obtained from the American Type Culture Collection (ATCC, Manassas, VA, USA), encompassing BT474, MCF 10A, a human breast cancer cell line, and 4T1, a mouse breast cancer cell line. The cells were cultured in a temperature-controlled incubator maintained at 37°C, under conditions of high humidity and a CO2 concentration of 5% (40).

### Culture of *Pseudoalteromonas elyakovii*

The *Pseudoalteromonas elyakovii* strain BMZ114943 was obtained from Mingzhoubio (Ningbo) and cultivated under strictly aerobic conditions using 2216E Liquid Medium (HB0132-1) (41).

### 5R 16S rRNA sequencing

The analysis process of 5R 16S sequencing involved the utilization of the Short MUltiple Regions Framework (SMURF) data analysis method (42). To facilitate taxonomic classification and estimate relative abundance, an optimized version of the Greengenes database (May 2013 version) was utilized in this investigation to identify the most probable set of 16S sequences.

### qRT-PCR quantification

For the quantification of qRT-PCR, we utilized the Tissue RNA Purification Kit Plus (ES Science, catalog no. RN002plus) for total RNA extraction from human tissue. The comparative Ct (2^−ΔΔCt^) approach was utilized to quantify the relative transcript levels of the genes of interest. The necessary primers are provided in Table S1.

### Single-cell RNA-sequencing

Following the guidelines provided by the producer, the Chromium Controller from 10× Genomics was utilized to handle individual cellular samples. We carried out profiling of gene expression at the single-cell level, focusing on the 3′ end of the genes. We utilized the Normalize Data function to standardize the raw data and identified genes exhibiting significant variation using the Find Variable Features function. Ultimately, we successfully identified and characterized each cell type based on its distinct gene expression profile.

### Double immunofluorescence staining combined with SweAMI probe *in situ* hybridization

Clinically obtained tissue samples were rapidly frozen using OCT embedding agent, then sectioned into 6 µm thick slices using a freezing microtome. Frozen sections were prepared according to the instructions. The slides were then examined using an Olympus#BX53 fluorescence microscope. The images were visualized using CaseViewer version 2.4 (3DHISTECH Ltd). Table S2 provides comprehensive information on the antibodies used in this study.

### Cell survivability assays

To evaluate the cell viability, the Cell Counting Kit-8 (CCK-8), a product of Topscience located in Shanghai, China, was utilized. This assessment was conducted in relation to the supernatant derived from *Pseudoalteromonas elyakovii*. The assessment of cell line vitality following exposure to bacterial supernatant was conducted utilizing the Calcein/PI Cell Viability/Cytotoxicity Detection Kit. The EVOS M7000 microscope (Thermo Fisher Scientific, USA) was employed to visualize viable cells. The calculation of the proportion of viable cells was performed using ImageJ software.

### Transwell migratory assay

The cell migration experiments were conducted using a transwell chamber (Corning, NY, USA) equipped with an 8 µm pore size filter. Within a 24-well transwell plate, the upper chamber was populated with 2 × 10^4^ cells that had been treated, suspended in 100 µL of serum-free medium (SFM).

Micrographs of the plates and migrating cells were acquired utilizing an EVOS M7000 microscope (Thermo Fisher Scientific, USA) and subsequently analyzed with ImageJ software.

### Wound healing assay

The cell density was standardized to 5 × 10^5^ per well and subsequently cultured in 6-well plates. When the cell confluence reached approximately 80%–90%, we gently scraped the cell monolayers using a sterile pipette tip with a volume of 200 µL. Utilizing Image J software, which is supplied by the National Institutes of Health (NIH) based in the United States, the photographic documentation of the samples was executed at both the 0 h mark and after a 24 h interval.

### *In vivo* allograft mouse model

Mice, all female BALB/c from QiZhen, Hangzhou, China, aged between 4 and 6 weeks, were administered an intravenous injection of 4T1 cells, specifically 5 × 10^6^ cells suspended in 50 µL PBS, directly into the inguinal fat pad of their mammary glands.

### Statistical analysis

The diversity of alpha was assessed using the test of Wilcoxon signed-rank and Kruskal−Wallis analysis. Utilizing the ANOSIM algorithm, an evaluation was carried out on the β-diversity PCA and PCoA analysis maps derived from both healthy and tumorous tissue samples. The dissimilarities in microbiota compositions among various samples were evaluated using the Wilcoxon signed-rank test; furthermore, the outcomes are presented in terms of the average value accompanied by its standard deviation, denoted as the mean ± SEM. The analysis of statistics involved the utilization of either a *t*-test or chi-square test. Analysis of the data set was performed utilizing GraphPad Prism Software, version 10.1.2, provided by GraphPad Prism Software Inc. The criterion for statistical significance was established at a *P*-value threshold below 0.05.

## Data Availability

The scRNA-seq data that support the findings of this study are openly available in the National Center for Biotechnology Information under accession number PRJNA1240614. The 16s rRNA data that support the findings of this study are openly available in the National Center for Biotechnology Information under accession number PRJNA1240197.

## SUPPLEMENTAL MATERIAL

Supplemental Material mbio.03873-24-s0001.docx Fig. S1 and S2, Tables S1 to S12, and additional experimental details.

ASM does not own the copyrights to Supplemental Material that may be linked to, or accessed through, an article. The authors have granted ASM a non-exclusive, world-wide license to publish the Supplemental Material files. Please contact the corresponding author directly for reuse.
