# Supplementary material for: Tumor-colonizing Pseudoalteromonas elyakovii metabolically reprograms the tumor microenvironment and promotes breast ductal carcinoma
Source: mBio. 2025 Apr 7;16(5):e03873-24. doi: 10.1128/mbio.03873-24 (PMC12077203; doi:10.1128/mbio.03873-24)
Supplement: Supplemental Material — Fig. S1 and S2, Tables S1 to S12, and additional experimental details. [file mbio.03873-24-s0001.docx]

**Supplemental Materials**

**Tumor-colonizing *Pseudoalteromonas elyakovii* metabolically reprograms the tumor microenvironment and promotes breast ductal carcinoma**

Shuyan Liu^1 2 3#^,Youpeng Pan^1 2 3#^,Chaopeng zheng^1 2 3#^ ,Qinghui Zheng ^1 2^,Yaoqiang Du^4^,Yajuan Zheng^1 2^ ,Hongchao Tang^1 2^,Xiaozhen Liu^1 2^,Jiancheng Mou^1 2 3^ ,Xin Zeng^1 2^,Zhuotao Yang^1 2 3^,Wenjuan Gui^1 2 6^,Yuning Tang^1 2 3^,Mingxing Xu^1 2 3^,Zhihao Ye^1 2 7^,Haotian Su^1 2 6^,Qiuran Xu^5*^ ,Xuli Meng^8*^

1.General Surgery, Cancer Center, Department of Breast Surgery, Zhejiang Provincial People's Hospital (Affiliated People's Hospital), Hangzhou Medical College, Hangzhou 310014 Zhejiang, China.

2.Key Laboratory for diagnosis and treatment of upper limb edema and stasis of breast cancer,Hangzhou 310014 Zhejiang, China.

3.The Second Clinical Medical College, Zhejiang Chinese Medical University, Hangzhou 310053 Zhejiang, China.

4.Laboratory Medicine Center, Zhejiang Provincial People's Hospital (Affiliated People's Hospital, Hangzhou Medical College), Hangzhou 310014 Zhejiang, China.

5. Zhejiang Key Laboratory of Tumor Molecular Diagnosis and Individualized Medicine, Zhejiang Provincial People's Hospital, Affiliated People's Hospital, Hangzhou Medical College, Hangzhou, 310014, China. Electronic address: xuqiuran@hmc.edu.cn.

6. The Second School of Clinical Medicine，Hangzhou Normal University, Hangzhou ,311121,

Zhejiang, China.

7. Wenzhou Medical University, Hangzhou, 325035, Zhejiang, China.

8.General Surgery, Cancer Center, Department of Breast Surgery, Zhejiang Provincial People's Hospital (Affiliated People's Hospital), Hangzhou Medical College, Hangzhou,310014, Zhejiang Province, China; Key Laboratory for diagnosis and Treatment of Upper Limb Edema and Stasis of Breast Cancer, Hangzhou, 310000 Zhejiang Province,China.Electronic address: [mxlmail@126.com](mailto:mxlmail@126.com)

*Correspondence should be addressed to Qiuran Xu([xuqiuran@hmc.edu.cn](mailto:xuqiuran@hmc.edu.cn)) and Xuli Meng(mxlmail@126.com)

# Shuyan Liu, Youpeng Pan and Chaopeng Zheng contributed equally to this article. Author order was determined based on their contributions. The authors declare no conflict of interest.

**Supplementary Table S1. Sequences of the primers used for qRT-PCR.**

**Supplementary Table S2. Reagents and antibodies.**

**Supplementary Table S3. Patient Demographics and Clinical Characteristics of BC Study Volunteers in the scRNA-seq survey.**

**Supplementary Table S4. The percentage of cells belonging to each cell type within a given sample is indicated.**

**Supplementary Table S5. Proportion of cell counts attributed to different samples in each cell type.**

**Supplementary Table S6. The top GO terms enriched in Epithelial cells.**

**Supplementary Table S7.The top GO terms enriched in Endothelial cells.**

**Supplementary Table S8. Pseudotime trajectory analysis of B cells.**

**Supplementary Table S9. Number of intercellular interactions.**

**Supplementary Table S10. Intensity of intercellular interactions.**

**Supplementary Table S11. GSVA analysis comparing three breast ductal carcinoma sample-specific enrichment pathways.**

**Supplementary Table S12.Proportion of microbial abundance in each cell type in each sample.**

**
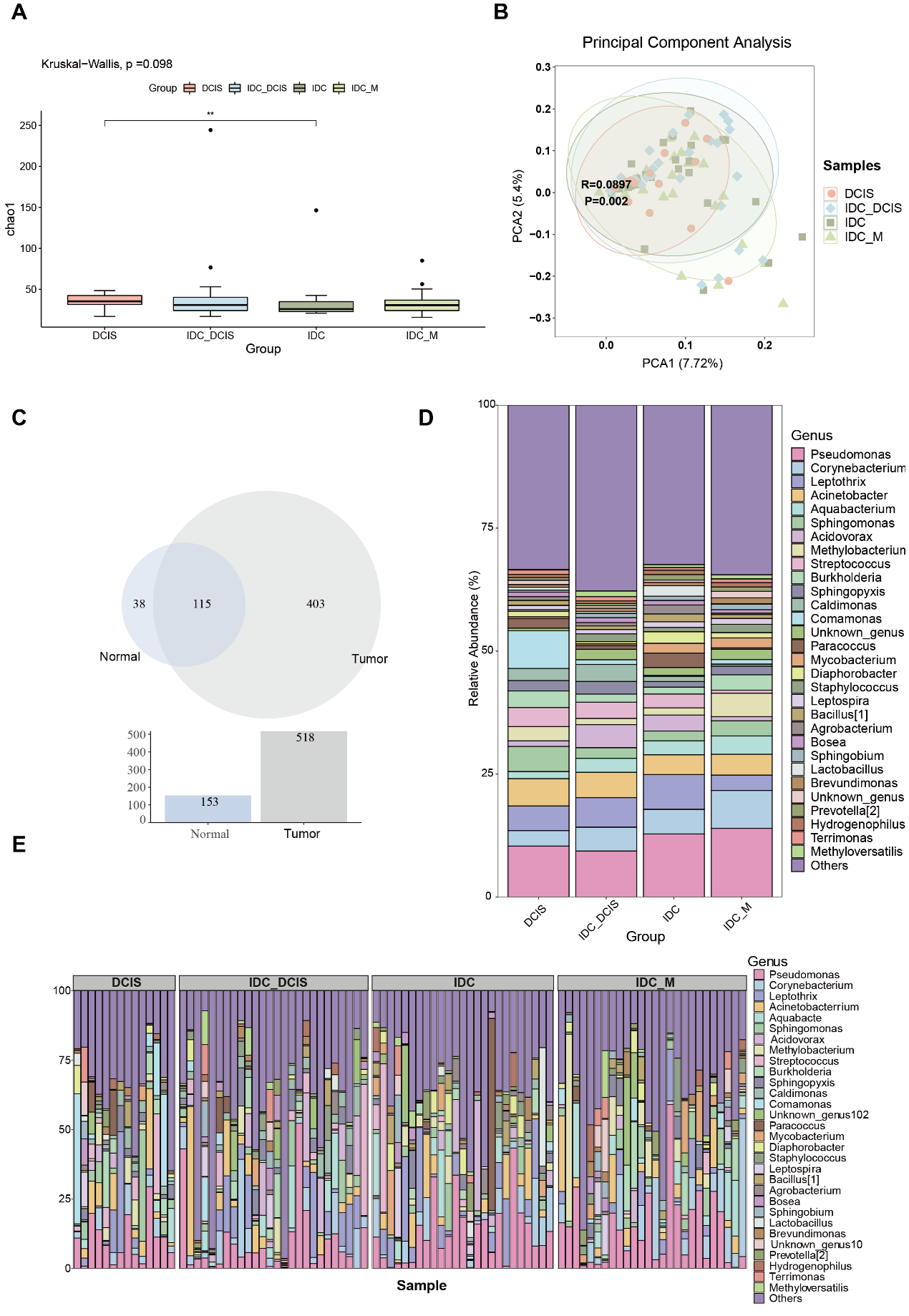
** Figure S1 Compositional analysis of the microbiota at different stages of ductal carcinoma of the breast. (A)Alpha diversity index of the intratumoral microbiota in various stages of ductal carcinoma of the breast (DCIS, IDC_DCIS, IDC, IDC_M);(B) Beta diversity index of microbiota composition among different stages of ductal carcinoma of the breast (DCIS, IDC_DCIS, IDC, IDC_M);(C) The relationship between tumor and paraneoplastic microbiota at the "Genus" level; (D) Proportional bar graphs depicting the intratumoral microbial composition of each group of samples at the "Genus" level;(E)Characterization of the composition of various individual microbiota in the ductal breast cancer group at the "Genus" level. Different color shades within the "Genus" represent microbes at distinct "Genus" levels.

**
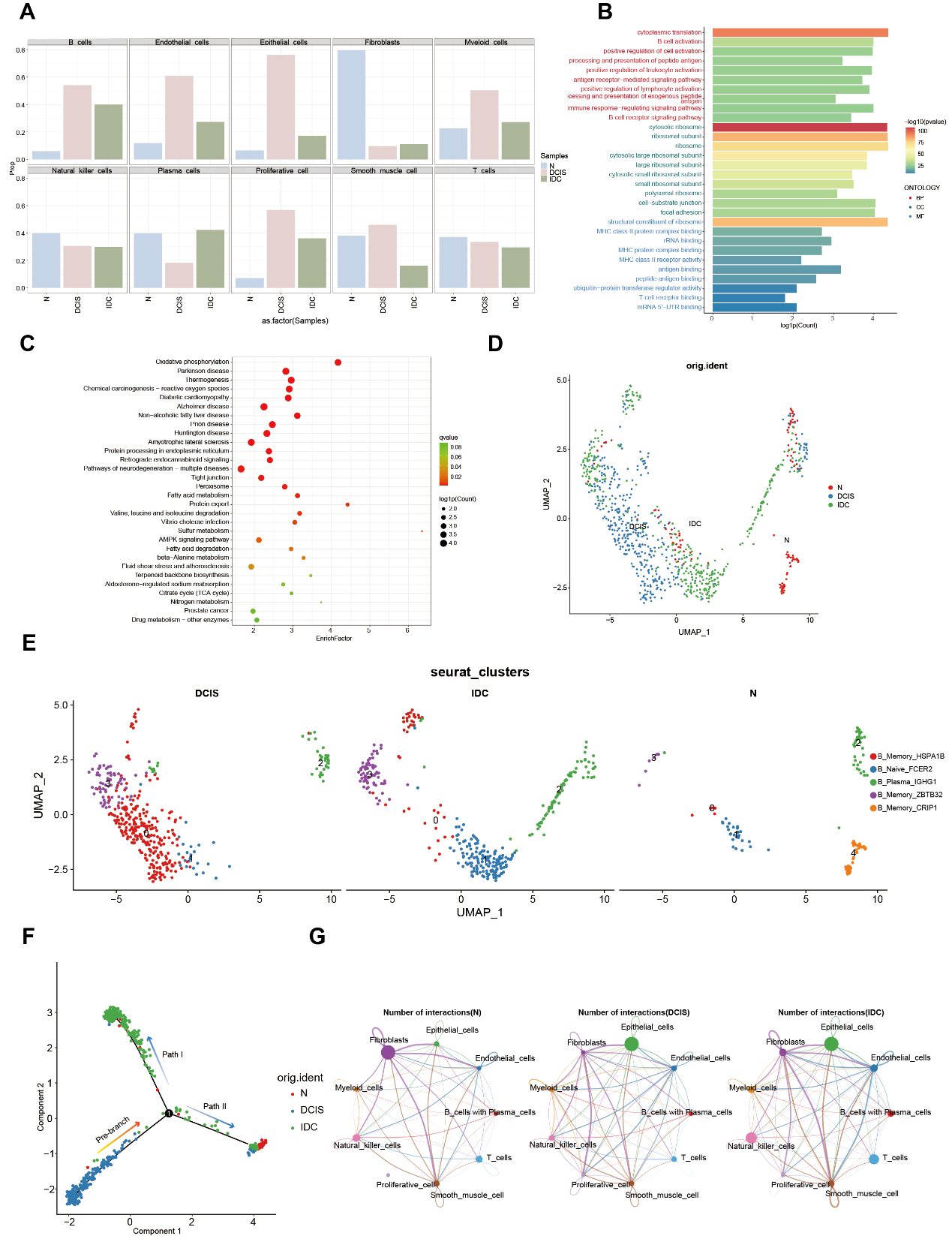
**

Figure S2 Investigation into scRNA-seq in Ductal Carcinoma of the Breast. (A) Proportion of cell counts attributed to different samples in each cell type. (B-C) The top GO terms enriched in B cells. The top KEGG terms enriched in Epithelial cells. The intensity represents the adjusted p - value of each hallmark. The dot size indicates the gene count for each hallmark. The Wilcoxon signed - rank test was employed to assess the difference. (D) UMAP visualization of three samples.(E) UMAP visualization of 5 B cell clusters in diverse samples. (F) Pseudotime trajectory of B cells and the distribution of different samples in the trajectory map. (G) The number of interactions among major cell types in various samples.

**Supplementary Methods**

**Patient recruitment and sample procurement**

The collection of samples and clinical data was conducted with the informed consent of participants, and the protocol (No. KT2023065) was approved by the Ethics Committee of Zhejiang Provincial People's Hospital. Between March 23, 2023 and June 4, 2024, a total of 91 patients were enrolled in this study. Among them, there were 11 cases of ductal carcinoma in situ (n_DCIS_=11) and an equal number of infiltrating ductal carcinoma with concurrent ductal carcinoma in situ (n_IDC_DCIS_=26). Additionally, there were 25 cases of infiltrating ductal carcinoma (n_IDC_=25) and another group comprising infiltrating ductal carcinoma with distant metastasis (n_IDC_M_=26). The paracancerous samples corresponding to patients diagnosed with ductal carcinoma in situ amounted to a total of 11(n_DCIS_=11). The study enrolled a total of 91 breast cancer patients, and their demographic characteristics including age, tumor stage, tumor molecular subtypes, lymph node involvement, and histological grade were meticulously documented. Patients with concurrent malignancies or those who had received antibiotic treatment within the preceding two months for newly diagnosed breast cancer were excluded from this analysis. These patients had previously undergone extensive surgical resection without receiving any additional adjuvant therapy. To account for potential intraoperative contamination, meticulous efforts were made to meticulously select uncontaminated tissue specimens. These carefully chosen samples were promptly transported to the laboratory and cryopreserved at -80°C for subsequent analysis.

**Cell lines and cell culture**

The cell lines utilized in this investigation were obtained from the American Type Culture Collection (ATCC, Manassas, VA, USA), encompassing BT474, MCF 10A, a human breast cancer cell line, and 4T1, a mouse breast cancer cell line. 4T1 cells were cultured in a growth medium comprising 10% fetal bovine serum (FBS) and 1% penicillin-streptomycin solution (PS) obtained from Thermo Fisher Scientific (catalog number 10378016). Conversely, BT474 cells were maintained in RPMI-1640 medium supplemented with 10% FBS, 1% PS, and human insulin at a concentration of 10 µg/mL. Furthermore, human normal mammary epithelial cells (MCF-10A) were cultured in a specialized medium. The cells were cultured in a temperature-controlled incubator maintained at 37°C, under conditions of high humidity and a CO2 concentration of 5%(1).

**Culture of** ***Pseudoalteromonas elyakovii***

The *Pseudoalteromonas elyakovii* strain BMZ114943 was obtained from Mingzhoubio (Ningbo) and cultivated under strictly aerobic conditions using 2216E Liquid Medium (HB0132-1). *Pseudoalteromonas*, a member of the *Gammaproteobacteria* class, is a prominent constituent within the Marine biota. It can be observed in diverse non-geothermal Marine ecosystems as an integral part of the *Alternomonas* order. The common characteristics observed in all members belonging to the *Pseudopanthomonas* genus include the requirement of Na+, a Gram-negative cell wall structure, rod-shaped cellular morphology, utilization of polar or unsheathed lateral flagella with a sheath for locomotion, and reliance on strict aerobic conditions for their chemically heterotrophic metabolism. The non-model strain *Pseudoalteromonas elyakovii*, originally discovered in extreme environments, is primarily employed for taxonomic classification, analytical identification, and aquaculture applications. In 1997, Ivanova et al. initially reported this strain based on their research in the field of microbial biotechnology. It was named after G.B. Elyakov(2).

**5R 16s rRNA sequencing**

The analysis process of 5R 16S sequencing involved the utilization of the Short MUltiple Regions Framework (SMURF) data analysis method(3). This approach integrated and examined the sequences from five amplified regions to taxonomically identify bacteria. Considering the low biomass of microorganisms in tumors, the presence of bacteria within tumors can be significantly influenced by the introduction of microorganisms from the surrounding environment, as well as during sample collection, DNA extraction, and PCR amplification procedures. The TMB algorithm was employed to eliminate potentially contaminated bacteria(4), in order to accomplish this objective. Consequently, the identification of bacteria present as contaminants at both the sampling and experimental ends was determined based on their prevalence in the negative control samples. In this study, a threshold of 50% prevalence was established to ascertain the presence of contaminating bacteria. According to the filtered flora data, we performed diversity analysis and differential identification of flora within and between groups. The SMURF algorithm was utilized for analyzing the 5R 16S sequencing data, while the Expectation-maximization algorithm was employed for bacterial identification in the samples and calculation of their relative abundance. To facilitate taxonomic classification and estimate relative abundance, an optimized version of the Greengenes database (May 2013 version) was utilized in this investigation to identify the most probable set of 16S sequences.

**qRT-PCR quantification**

For the quantification of qRT-PCR, we utilized the Tissue RNA Purification Kit Plus (ES Science, catalog no. RN002plus) for total RNA extraction from human tissue. Subsequently, the High-Capacity cDNA Synthesis Kit for Reverse Transcription (ABclonal, catalog no.RK20433) was employed for cDNA synthesis. A reaction mixture of 20 μL containing SYBR Green Master Mix (2X; ABclonal,catalog no.RK21203), forward primer at a concentration of 10 μmol/L, reverse primer at a concentration of 10 μmol/L, and 1 μL sample DNA was loaded onto the Applied Biosystems 7500 Real-Time PCR System (Thermo Fisher Scientific). The qRT-PCR reaction was performed according to the subsequent protocol:an initial step of denaturation at 95°C for 3 minutes, followed by a series of 40 cycles consisting of denaturation at 95°C for 5 seconds and annealing extension at 60°C for 34 seconds. The experiment concluded with the generation of a dissociation curve. Bacterial load was determined by comparing threshold cycle (Ct) values against a standard curve derived from analyzing the abundance of bacteria using the genetic marker, specifically the 16s rRNA. The experiments were conducted using a minimum of three biological replicates. The comparative Ct (2^-ΔΔCt^) approach was utilized to quantify the relative transcript levels of the genes of interest. The necessary primers are provided in Table S1.

**Single-cell RNA-sequencing (scRNA-seq)**

The samples were finely minced into 1mm^3^ fragments using a sterile scalpel. Subsequently, they were mixed with 5ml of digestion buffer composed of Collagenase type I (Sigma, C0130) at a concentration of 2mg/mL, Collagenase type II (Sigma, C6885) at a concentration of 2mg/mL, and DNase I (Worthington, LS006344) at a concentration of 200U/ml in RPMI medium (Coring,10-040-CV). The mixture was incubated in an environment maintained at 37℃ with continuous agitation for 45 minutes. Following this step, the suspension was filtered through a mesh with pores measuring 100μm (Falcon,3523260), followed by centrifugation under refrigeration conditions set to approximately 4℃ for 10 minutes at a force of 400g. The cells that had been pelleted were then redispersed within a lysis buffer specifically designed for the removal of red blood cells (Solarbio, R1010) and incubated for 2 minutes. Subsequently, the resulting suspension was filtered through a 40μm filter (Falcon, 3523240), followed by collection via centrifugation at 400g for 10 minutes at 4℃. Afterwards, the cells were resuspended in PBS (BI, 02-024-1ACS) supplemented with BSA at a concentration of 0.04% (Sigma, B2064). Manual cell counting was performed using Trypan blue staining (Thermo, T10282) and AO-PI staining (LUNA,D23001) after centrifugation at 400g for 10 minutes at 4℃ and subsequent resuspension. Following the guidelines provided by the producer, the Chromium Controller from 10X Genomics was utilized to handle individual cellular samples.

We carried out profiling of gene expression at the single-cell level, focusing on the 3' end of the genes. The cell suspension was introduced using the Chromium single cell controller (10x Genomics) following the guidelines provided by the producer, resulting in the generation of gel beads that encapsulated individual cells within an emulsion. The cells that were captured were subjected to lysis to release their RNA. This RNA was subsequently reverse-transcribed to incorporate unique barcodes within separate Gel Bead-in-Emulsion (GEM) particles. 3'gene expression libraries with cell-specific barcodes were sequenced.

The initial sequence reads were mapped to the human reference genome, specifically the refdata-gex-GRCh38-2020-A, employing Cell Ranger count version 7.0.0 with the preset settings. The digital expression matrix was derived from the 'filtered_feature_bc_matrix' output directory, which was produced by the Cell Ranger count software. We utilized the Doublet Finder algorithm to detect doublets in single-cell RNA sequencing data, subsequently removing duplicated cells. The identification of distinct clusters and signature genes was performed using the Seurat R package (v4.1.1). Briefly, genes expressed in fewer than three cells were excluded, and only cells with unique feature counts ranging from the 300th to the 97.5th percentile were included for further analysis, ensuring that mitochondrial expression remained below 25%. We utilized the Normalize Data function to standardize the raw data and identified genes exhibiting significant variation using the Find Variable Features function. Subsequently, we conducted data integration by employing canonical correlation analysis to pinpoint common factors influencing variation across multiple datasets. This integration process involved utilizing the Select Integration Features, Find Integration Anchors, and Integrate Data functions. The data underwent linear transformation and principal component analysis, utilizing the Scale Data and Run PCA functions, respectively, based on genes exhibiting high variability.

Relevant genes exhibiting high variability (top 3000) were selected for PCA. Cluster analysis was performed using the Find Neighbors and Find Clusters methods, considering the 20 most influential principal components. The clustering parameter employed was "resolution=0.3". The groupings of data were visualized employing both the UMAP and t-SNE methods. The Seurat Find Markers function was employed to identify signature genes that characterize each cluster, cell category, and subcategory. This function utilized the Wilcoxon rank-sum test was applied to assess differences in gene expression between cells belonging to a specific cluster, cell type, or subgroup and those from other groups. Ultimately, we successfully identified and characterized each cell type based on its distinct gene expression profile. The identification of cell clusters was performed using distinct marker genes associated with well-characterized cell types, including epithelial cells, plasma cells, myeloid cells, natural killer (NK) cells, B cells, T cells, smooth muscle cells, proliferative cells, fibroblasts and endothelial cells(5).

The software tools Cluster Profiler and Seurat were employed to perform GO enrichment and KEGG enrichment analyses on cluster markers. The Benjamini-Hochberg procedure was employed to make adjustments for multiple comparisons. The marker genes underwent wilcox test and log-scale fold change analysis (with a threshold of logfc ≥ 0.25). Visualization of the results was accomplished by utilizing an R package.

**Double immunofluorescence staining** **combined with SweAMI probe in situ hybridization**

Clinically obtained tissue samples were rapidly frozen using OCT (Optimal Cutting Temperature) embedding agent, then sectioned into 6μm thick slices using a freezing microtome. The sections were then adhered to glass microscope slides and allowed to dry in the air for a duration of 30 minutes at ambient temperature. Subsequently, the cell slides or frozen sections were immersed in 0.1mol/L citrate buffer for 10 minutes at room temperature to rehydrate the tissue cells. The cells were then treated with the drilling solution for 10 minutes at room temperature, followed by three rinses with PBS (phosphate-buffered saline) of 0.1mol/L concentration. Finally, the sections underwent washing with PBST (phosphate-buffered saline containing Tween-20) diluted to a concentration of 0.1%. The excess liquid on the tissue was removed, and an appropriate amount of proteinase K (5 μg/mL Shengon B900004-0100) working solution was applied onto the tissue. Subsequently, the tissue was incubated at 37℃ for 25 minutes, this was succeeded by a trio of rinses using a solution of phosphate-buffered saline (PBS). After gentle drying, the tissues were covered with pre-hybridization mixture that includes DNA from salmon testes to enhance hybridization efficiency (the ultimate concentration of DNA derived from salmon sperm was specified at 100 μg/mL) and incubated in a humidified chamber at 37℃ for 1 hour. Finally, the specialized cover glass for in situ hybridization was carefully placed over the tissues. After the coverslips were taken off, the samples underwent three successive washes with PBS at ambient temperature, with each wash lasting for a period of 5 minutes. The surplus fluid from the tissue was removed, followed by the application of the hybridization solution. The 100μm probe (directly labeled probe) was diluted with the hybridization solution to achieve the corresponding working concentration (1:50). Each slide received 100μl of hybridization solution, and a cover glass was placed over the tissues for in situ hybridization. Subsequently, it was sealed using adhesive for four weeks and incubated overnight in a moist chamber at 42℃. Following this, the coverslips were detached and subjected to a wash step using 2×SSC at 40°C for 5 minutes. The cells underwent further washing in 1×SSC at 40°C for another period of 5 minutes. A subsequent wash with PBS at 40°C lasting an additional duration of 5 minutes followed suit. To block any non-specific binding sites, the cells were treated with a blocking solution containing donkey serum (10%) for half an hour at room temperature. After removing the blocking solution, primary monoclonal antibodies against CD3 (1:200; Proteintech, Rabbit IgG Publications107), as well as CD20 (1:400; Proteintech, Mouse IgG2b Clone4A7G3), were co-incubated under ambient conditions for one hour before being incubated flat in a moist chamber overnight at 4°C. On the day that was the third in the sequence, the tissue sections were allowed to reach equilibrium at ambient temperature for a duration of 20 minutes. Subsequently, they underwent three rounds of rinsing with TBST solution, followed by another three rounds of rinsing with dip solution for a duration of 5 minutes each time. Next, we added 1μL of Alexa Fluor 488 donkey anti-mouse lgG(H+L) antibody stock solution and 1μL of Alexa Fluor 647 donkey anti-rabbit lgG(H+L) antibody stock solution to prepare a mixture. This mixture was then combined with 400 μL TBST and incubated at 37°C for 45 minutes. After the incubation period, the sections were removed from the oven and washed with TBST three times for 5 minutes each. Afterward, the sections were treated with DAPI staining reagent and then placed in a dark setting for a period of 8 minutes to allow for incubation. Following rinsing, an anti-fluorescence quenching sealing agent was utilized to seal the slices. The slides were then examined using an Olympus#BX53 fluorescence microscope. The images were visualized using CaseViewer version 2.4 (3DHISTECH Ltd). The Table S2 provides comprehensive information on the antibodies used in this study.

**Cell survivability assays**

To evaluate the cell viability, the Cell Counting Kit-8 (CCK-8), a product of Topscience located in Shanghai, China, was utilized. This assessment was conducted in relation to the supernatant derived from *Pseudoalteromonas elyakovii*. The 96-well plate was seeded with approximately four thousand cells per well and incubated for 24 hours within a regulated chamber that maintains a constant 5% CO2 atmosphere and steady humidity levels, the temperature was precisely set at 37°C (6). Following exposure to a range of concentrations from the supernatant of *Pseudoalteromonas elyakovii*, the cells underwent an incubation period totaling 24 hours. Post medium removal, each well received an addition of 100 microliters of a freshly prepared medium that was enriched with a 10% concentration of the CCK-8 viability reagent, followed by a 2-hour incubation period. A microplate reader was utilized to measure the optical density at the specific wavelength of 450 nm. Dose-response histograms were generated with GraphPad Prism 10.1.2 (GraphPad Inc., La Jolla, San Diego, CA, USA). The assessment of cell line vitality following exposure to bacterial supernatant was conducted utilizing the Calcein/PI Cell Viability/Cytotoxicity Detection Kit, which is a product of Beyotime, a Chinese company. The 4T1 and BT474 cell lines were resuspended within a medium enriched with serum at a density of 3 × 10^4^ cells and subsequently seeded into 24-well plates for a duration of one day. Afterwards, the staining process was conducted in a light-restricted environment using a solution containing 1mM Calcein AM and 1mM PI. The EVOS M7000 microscope (Thermo Fisher Scientific, USA) was employed to visualize viable cells exhibiting green fluorescence and non-viable cells displaying red fluorescence. The calculation of the proportion of viable cells was performed using ImageJ software.

**Transwell migratory assay**

The cell migration experiments were conducted using a transwell chamber (Corning, NY, USA) equipped with an 8 μm pore size filter. Within a 24-well transwell plate, the upper chamber was populated with 2×10^4^ cells that had been treated, suspended in 100 μL of serum-free medium (SFM). Concurrently, the lower chamber was replenished with a medium that included a 10% concentration of fetal bovine serum (FBS), amounting to 550 μL. Following a 24-hour incubation, the cells that had migrated were subjected to fixation using a 4% Paraformaldehyde Fix Solution (PFA), a process that lasted for a duration of 40 minutes. Following the fixation process, the cells underwent staining with a 0.1% crystal violet solution, a procedure that was carried out over the same period of time. After the staining procedure, the plates underwent a thorough rinse with phosphate-buffered saline (PBS). Subsequently, the residual cells present in the upper compartment were carefully eliminated with the aid of a sterile cotton swab. High-resolution imaging of the plates and the migrating cells was accomplished with the assistance of a sophisticated microscope from Thermo Fisher Scientific, a company based in the USA, specifically the EVOS M7000 model. The captured images were then analyzed quantitatively using Image J, a specialized imaging software for scientific research.

**Wound healing assay**

The cell density of 4T1 cells was standardized to 5×10^5^ per well and subsequently cultured in 6-well plates. When the cell confluence reached approximately 80% to 90%, we gently scraped the cell monolayers using a sterile pipette tip with a volume of 200 μL. Subsequently, any nonadherent cells were removed by thorough rinsing with PBS. In serum-free medium (SFM), cells were exposed to supernatant of different concentration gradients and then kept at 37°C for 24 h in a 5% CO2 incubator. Utilizing Image J software, which is supplied by the National Institutes of Health (NIH) based in the United States, the photographic documentation of the samples was executed at both the 0-hour mark and after a 24-hour interval. Subsequently, numerical analysis was conducted on the converted images to determine the wound width and percentage of healing for the scratch area.

**In vivo allograft mouse model**

A random allocation was conducted to divide the mice into two distinct groups, each comprising five individuals. These mice, all female BALB/c from QiZhen, Hangzhou, China, aged between 4 to 6 weeks, were administered with an intravenous injection of 4T1 cells, specifically 5×10^6^ cells suspended in 50 μL PBS, directly into the inguinal fat pad of their mammary glands. In addition to the conventional method where tumor cells are implanted into mice to form a control cohort, an experimental approach was taken where the tumor cells were subjected to treatment with the supernatant derived from *Pseudoalteromonas elyakovii*. The measurements of tumor volume and body weight in mice were recorded at 3-day intervals. The calculation for tumor volume involved dividing the product of length and the square of width by 2. The mice were euthanized upon completion of the treatment, and the tumors were excised for subsequent weight measurement. The animal protocols have received approval from institutional review boards in compliance with ethical guidelines (Ethics Number: 20241011725429).

**REFERENCES**

1. Zhu Z, Huang J, Li X, Xing J, Chen Q, Liu R, Hua F, Qiu Z, Song Y, Bai C, Mo YY, Zhang Z. 2020. Gut microbiota regulate tumor metastasis via circRNA/miRNA networks. Gut Microbes 12:1788891.

2. Sawabe T, Tanaka R, Iqbal MM, Tajima K, Ezura Y, Ivanova EP, Christen R. 2000. Assignment of Alteromonas elyakovii KMM 162T and five strains isolated from spot-wounded fronds of Laminaria japonica to Pseudoalteromonas elyakovii comb. nov. and the extended description of the species. Int J Syst Evol Microbiol 50 Pt 1:265-271.

3. Fuks G, Elgart M, Amir A, Zeisel A, Turnbaugh PJ, Soen Y, Shental N. 2018. Combining 16S rRNA gene variable regions enables high-resolution microbial community profiling. Microbiome 6:17.

4. Nejman D, Livyatan I, Fuks G, Gavert N, Zwang Y, Geller LT, Rotter-Maskowitz A, Weiser R, Mallel G, Gigi E, Meltser A, Douglas GM, Kamer I, Gopalakrishnan V, Dadosh T, Levin-Zaidman S, Avnet S, Atlan T, Cooper ZA, Arora R, Cogdill AP, Khan MAW, Ologun G, Bussi Y, Weinberger A, Lotan-Pompan M, Golani O, Perry G, Rokah M, Bahar-Shany K, Rozeman EA, Blank CU, Ronai A, Shaoul R, Amit A, Dorfman T, Kremer R, Cohen ZR, Harnof S, Siegal T, Yehuda-Shnaidman E, Gal-Yam EN, Shapira H, Baldini N, Langille MGI, Ben-Nun A, Kaufman B, Nissan A, Golan T, Dadiani M, et al. 2020. The human tumor microbiome is composed of tumor type-specific intracellular bacteria. Science 368:973-980.

5. Liu YM, Ge JY, Chen YF, Liu T, Chen L, Liu CC, Ma D, Chen YY, Cai YW, Xu YY, Shao ZM, Yu KD. 2023. Combined Single-Cell and Spatial Transcriptomics Reveal the Metabolic Evolvement of Breast Cancer during Early Dissemination. Adv Sci (Weinh) 10:e2205395.

6. Jin J, Qiu S, Wang P, Liang X, Huang F, Wu H, Zhang B, Zhang W, Tian X, Xu R, Shi H, Wu X. 2019. Cardamonin inhibits breast cancer growth by repressing HIF-1alpha-dependent metabolic reprogramming. J Exp Clin Cancer Res 38:377.
